# Supplementary figures and images for: Early correction of synaptic long-term depression improves abnormal anxiety-like behavior in adult GluN2B-C456Y-mutant mice
Source: PLoS Biol. 2020 Apr 30;18(4):e3000717. doi: 10.1371/journal.pbio.3000717 (PMC7217483; doi:10.1371/journal.pbio.3000717)

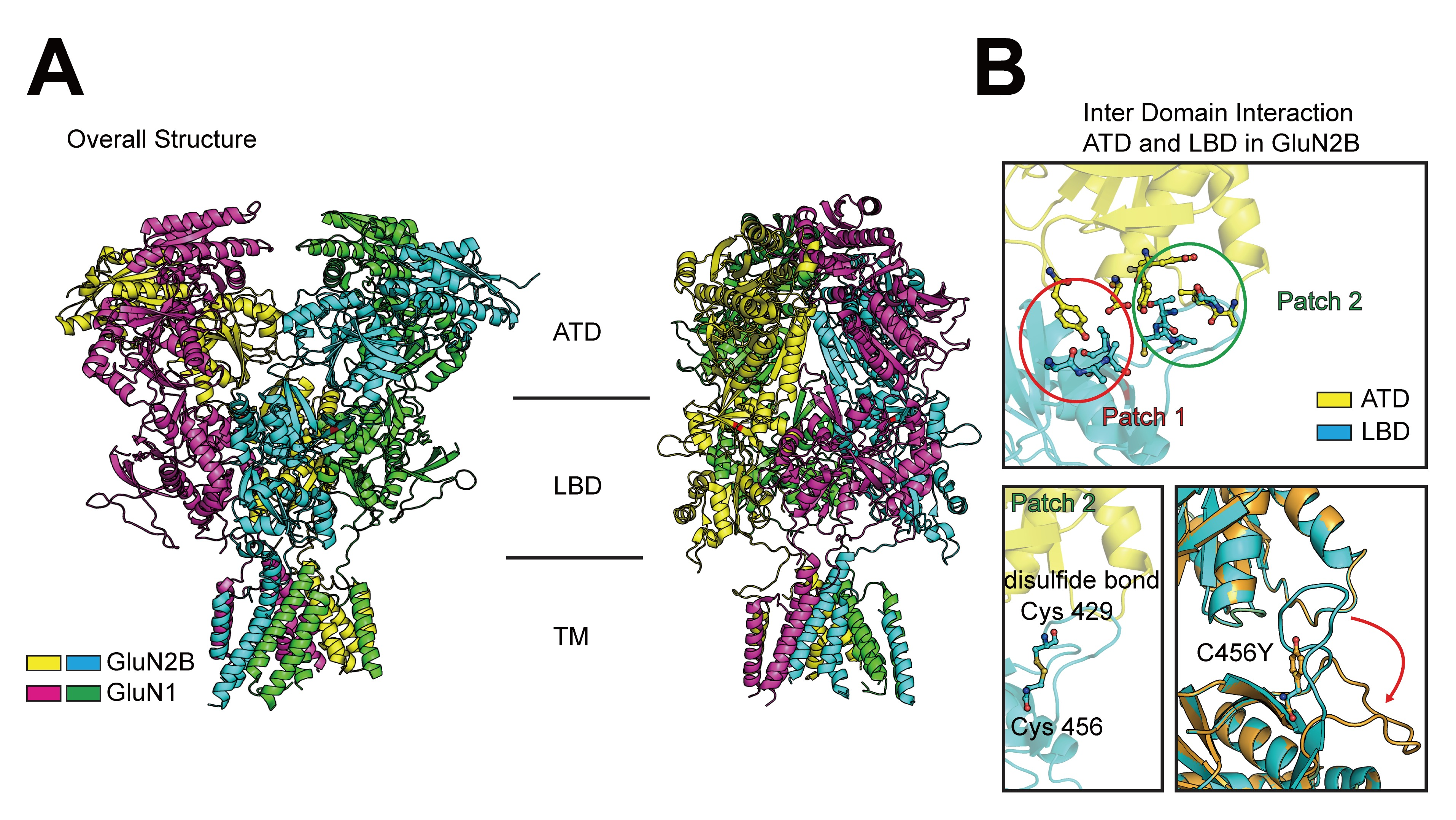

Supplement: S1 Fig — (A and B) Molecular modeling of the GluN2B-C456Y protein in complex with the GluN1 subunit of NMDARs. Note that the two cysteine residues in patch 1 of the LBD and patch 2 of the ATD in the WT GluN2B protein form a disulfide bond that strengthens the interaction between LBD and ATD, a bond that is disrupted by the GluN2B-C456Y mutation in patch 1 of the LBD. ATD, amino-terminal domain; LBD, ligand-binding domain; NMDAR, N-methyl-D-aspartate receptor; WT, wild type. (TIF) [file pbio.3000717.s001.tif]

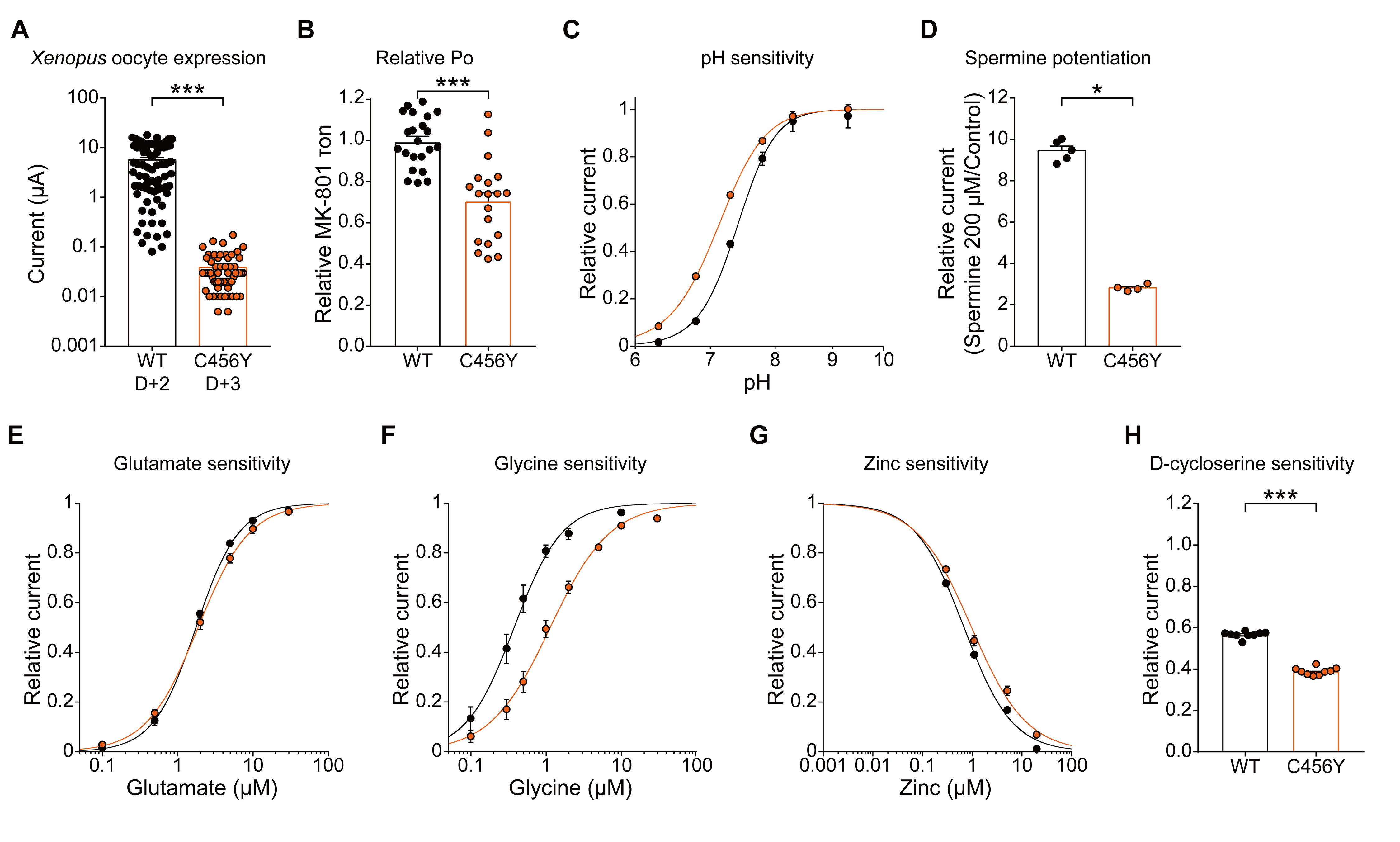

Supplement: S2 Fig — (A) The GluN2B-C456Y mutation strongly decreases diheteromeric GluN1/GluN2B NMDAR currents in Xenopus oocytes. Note that the amount of the mutant currents is <1% of the WT currents, despite the fact that mutant currents were recorded 1 day later than WT (3 and 2 days following oocyte injection, respectively). n = 73 oocytes for WT (5.70 ± 0.61 μA) and 59 oocytes for C456Y (0.039 ± 0.004 μA), ***P < 0.001, Mann-Whitney. (B) The GluN2B-C456Y mutation increases maximal open probability, as assessed by measuring MK-801 inhibition kinetics. n = 22 oocytes for WT (1 ± 0.03, relative τon) and 19 oocytes for C456Y (0.71 ± 0.05, relative τon), ***P < 0.001, Mann-Whitney. (C) The GluN2B-C456Y mutation reduces the sensitivity to extracellular protons. n = 4 oocytes for WT (pH IC50 = 7.49 ± 0.016) and 5 oocytes for C456Y (pH IC50 = 7.11 ± 0.0075), *P = 0.016, Mann-Whitney. (D) The GluN2B-C456Y mutation decreases the spermine-dependent potentiation. n = 5 oocytes for WT (9.45 ± 0.51, spermine potentiation) and 4 oocytes for C456Y (2.83 ± 0.077, spermine potentiation),*P = 0.016, Mann-Whitney. (E) The GluN2B-C456Y mutation does not affect the sensitivity to glutamate. n = 4 oocytes for WT (EC50 = 1.75 ± 0.04 μM) and 3 oocytes for C456Y (EC50 = 1.86 ± 0.02 μM), P = 0.23, Mann-Whitney. (F) The GluN2B-C456Y mutation decreases the sensitivity to glycine. n = 4 oocytes for WT (EC50 = 0.38 ± 0.017 μM) and 9 oocytes for C456Y (EC50 = 1.13 ± 0.049 μM), **P = 0.007, Mann-Whitney. (G) The GluN2B-C456Y mutation has minimal effect on the sensitivity to extracellular zinc. n = 11 oocytes for WT (IC50 = 0.68 ± 0.07 μM) and 11 oocytes for C456Y (IC50 = 0.97 ± 0.1 μM), ***P < 0.001, Mann-Whitney. (H) D-cycloserine is a partial agonist at GluN2B-C456Y mutant receptors. Currents recorded in 100 μM glutamate plus 100 μM D-cycloserine were normalized to currents recorded in 100 μM glutamate + 100 μM glycine (no D-cycloserine). n = 9 oocytes for WT (relative current: 0.57 ± 0.005) and 9 oocytes for C [file pbio.3000717.s002.tif]

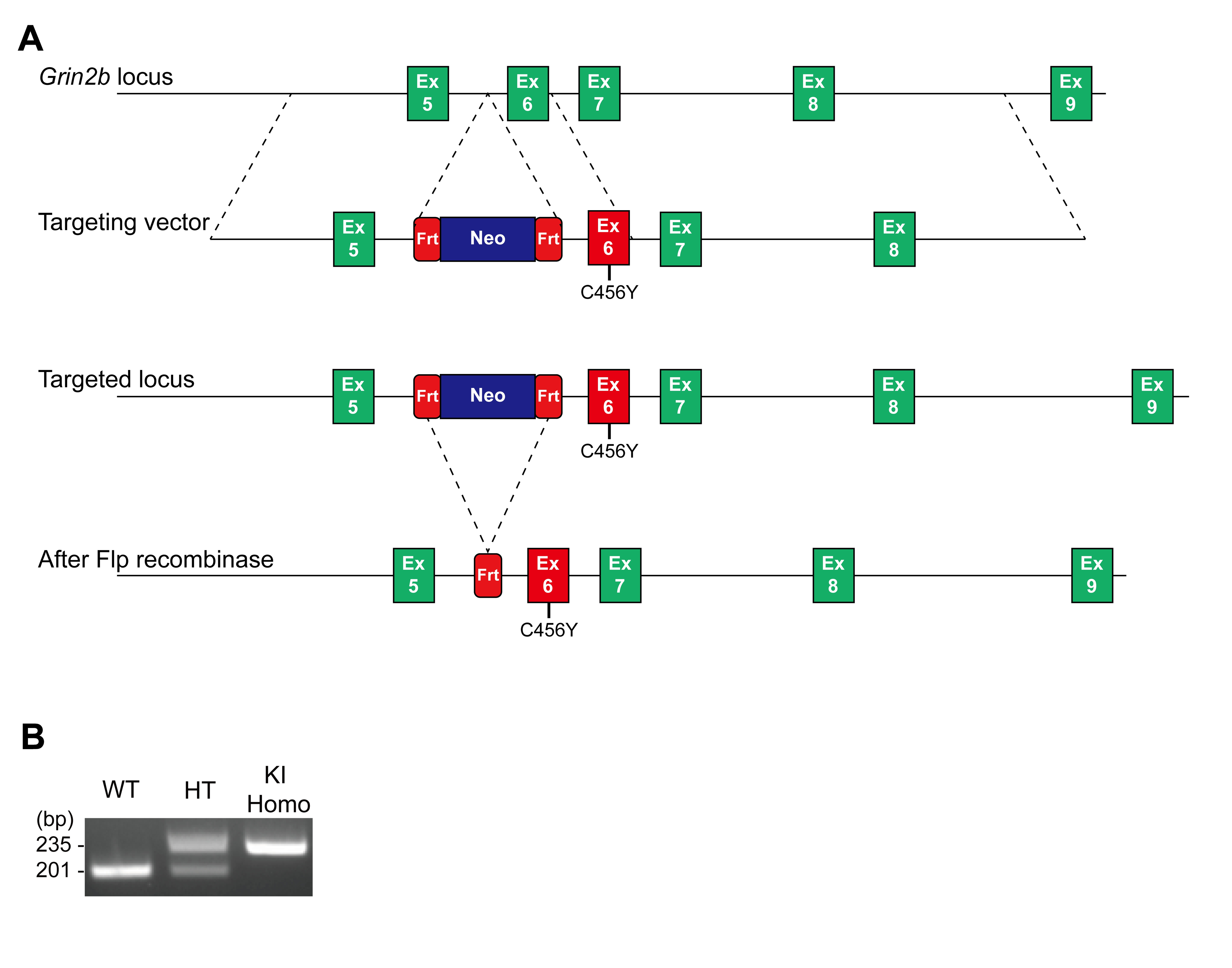

Supplement: S3 Fig — (A) Knock-in strategy for the GluN2B-C456Y mutation in mice. WT exon 6 was replaced with a mutant exon 6 containing the C456Y mutation. (B) PCR genotyping of homozygous (“Homo”) and HT KI mice. Ex, exon; Frt, flippase target site; Homo, homozygous; HT, heterozygous; KI, knock-in; Neo, neomycin gene; WT, wild type. (TIF) [file pbio.3000717.s003.tif]

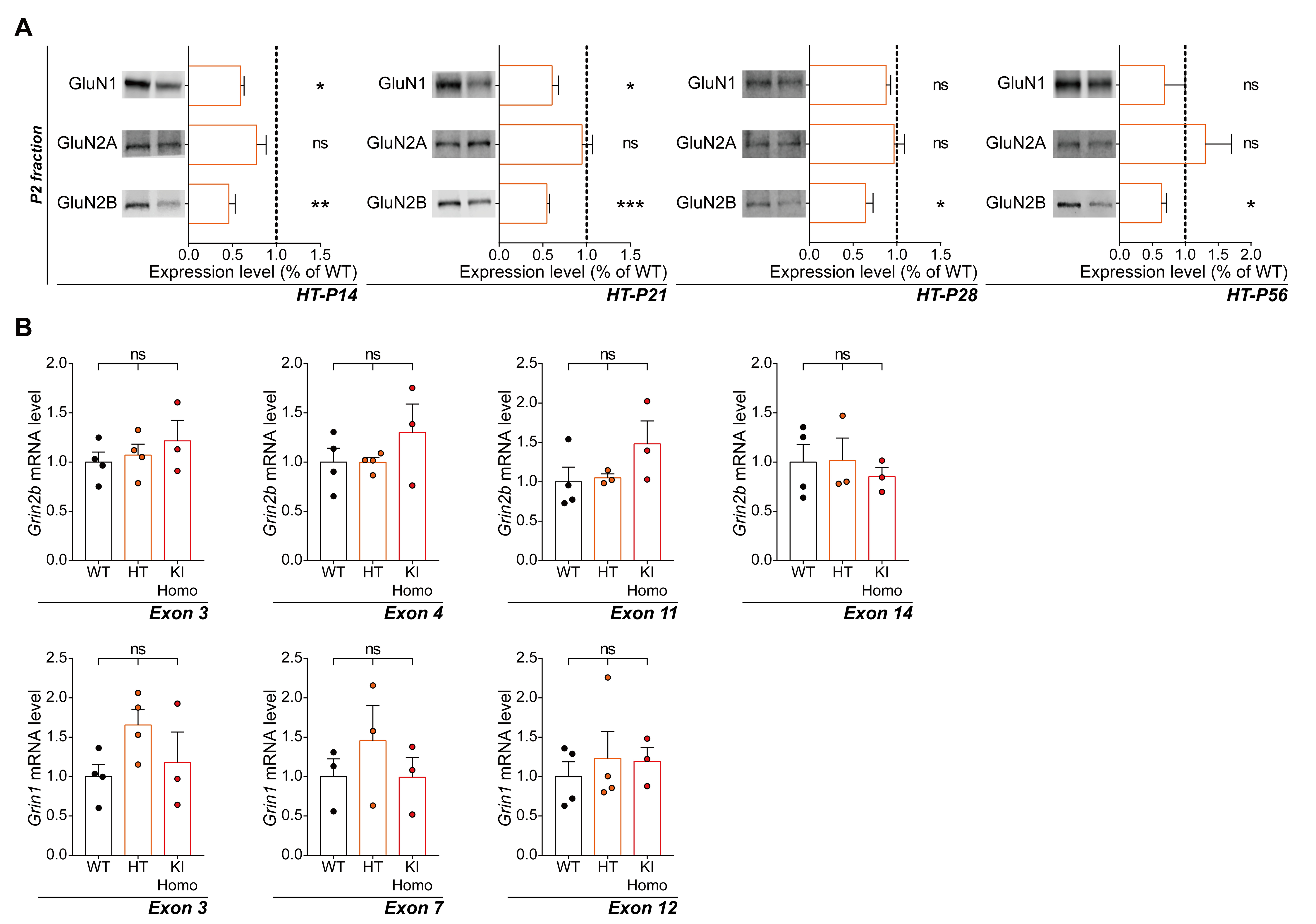

Supplement: S4 Fig — (A) Crude synaptosomal fractions from the Grin2b+/C456Y brain at multiple developmental stages (E20, P14, P21, P28, and P56) were immunoblotted with the indicated antibodies. For quantification (bar graphs), average levels of GluN1, Glu2A, and Glu2B proteins from Grin2b+/C456Y mice were normalized to those from WT mice. n = 4 mice for WT and HT, *P < 0.05, **P < 0.01, ***P < 0.001, Student t test. (B) Normal levels of Grin2b and Grin1 (encoding GluN1) mRNAs in WT, HT, and homozygous (“Homo”) KI embryos (E20), as indicated by the results of RT-qPCR reactions targeting Grin2b mRNA exons 3, 4, 11, or 14, and Grin1 mRNA exons 3, 7, or 12. n = 4 mice for WT, 4 for HT, and 3 for Homo, one-way ANOVA with Tukey’s test. The numerical data underlying this figure can be found in S3 Data. E, embryonic day; HT, heterozygous; KI, knock-in; ns, not significant; P, postnatal day; RT-qPCR, real-time quantitative PCR; WT, wild type. (TIF) [file pbio.3000717.s004.tif]

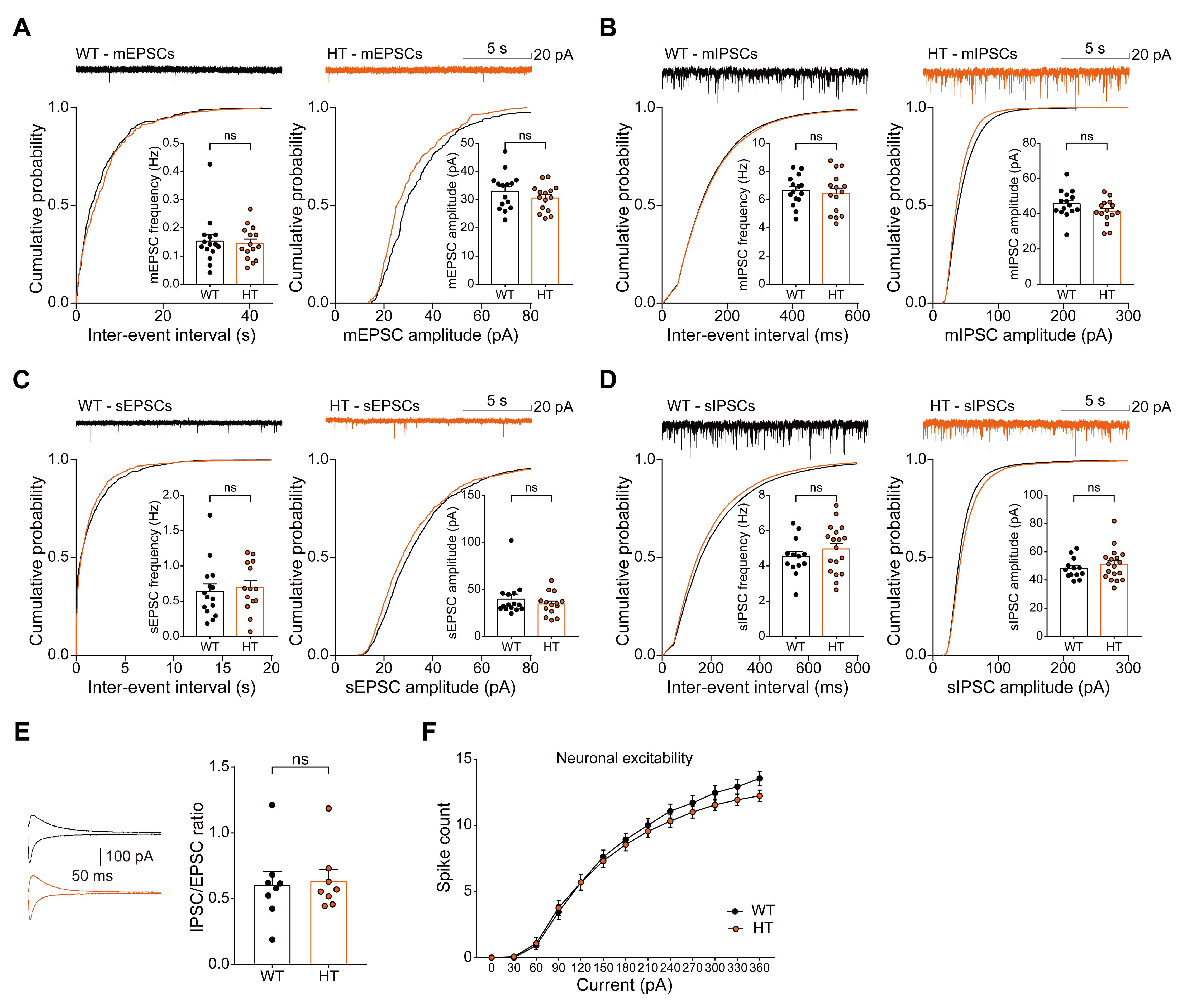

Supplement: S5 Fig — (A) Normal mEPSCs in CA1 neurons of Grin2b+/C456Y mice (P18–20). n = 15 neurons from 3 mice for WT and 15 (3) for HT, Mann-Whitney test (frequency) and Student t test (amplitude). (B) Normal mIPSCs in CA1 neurons of Grin2b+/C456Y mice (P21–23). n = 15 (3) for WT and HT, Student t test. (C) Normal sEPSCs in CA1 neurons of Grin2b+/C456Y mice (P22–24). n = 15 (3) for WT and 14 (4) for HT, Mann-Whitney test. (D) Normal sIPSCs in CA1 neurons of Grin2b+/C456Y mice (P22–24). n = 13 (3) for WT and 18 (4) for HT, Mann-Whitney test (frequency) and Student t test (amplitude). (E) Normal ratio of evoked IPSCs over evoked EPSCs in the CA1 region of Grin2b+/C456Y mice (P20–22). n = 8 (4) for WT and 8 (3) for HT, Mann-Whitney test. (F) Normal neuronal excitability in CA1 neurons of Grin2b+/C456Y mice (P21–23), as indicated by the current-firing relationship. n = 13 (3) for WT and 13 (4) for HT, two-way ANOVA. The numerical data underlying this figure can be found in S3 Data. EPSC, excitatory postsynaptic current; HT, heterozygous; IPSC, inhibitory postsynaptic current; mEPSC, miniature EPSC; mIPSC, miniature IPSC; ns, not significant; P, postnatal day; sEPSC, spontaneous EPSC; sIPSC, spontaneous IPSC; WT, wild type. (TIF) [file pbio.3000717.s005.tif]

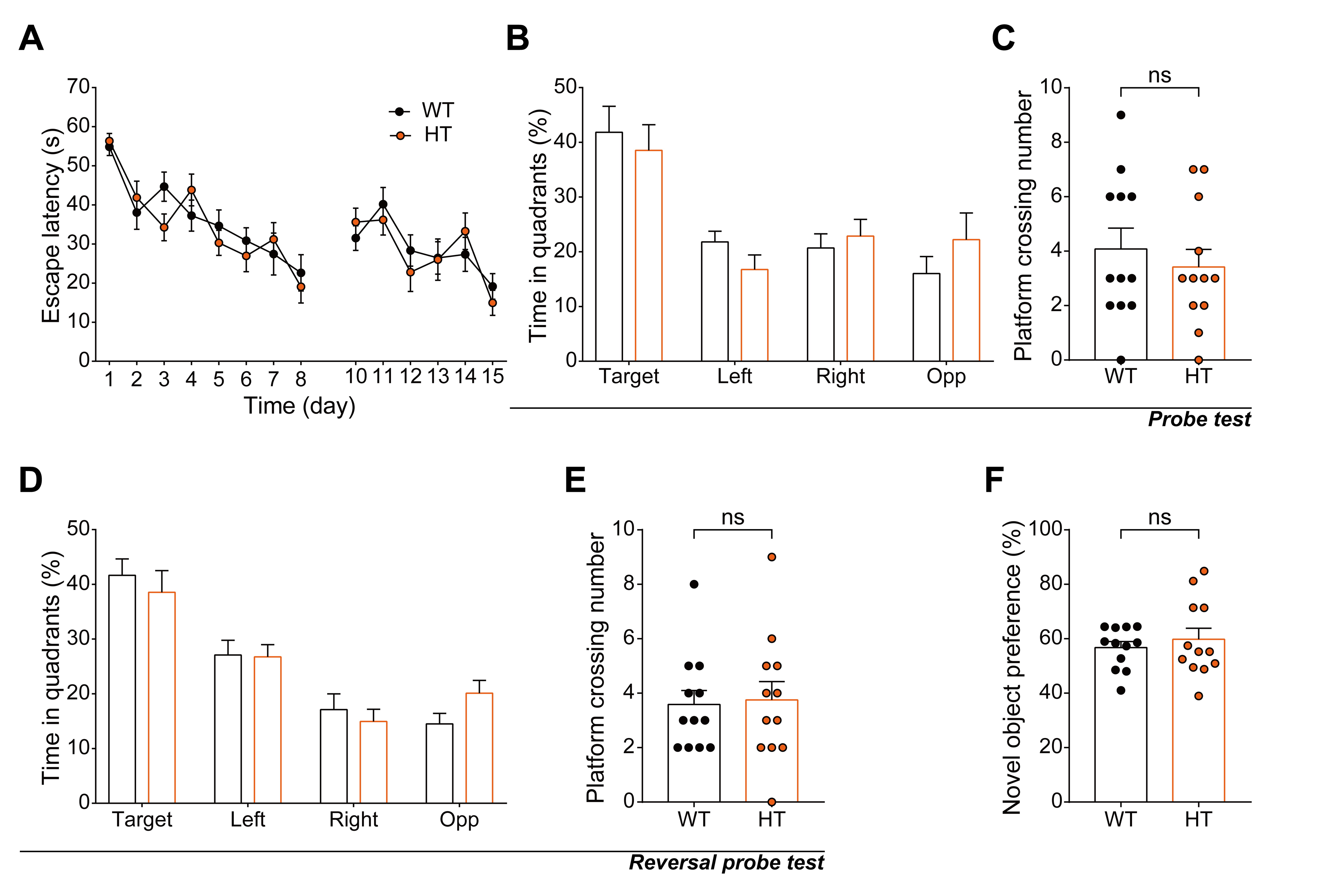

Supplement: S6 Fig — (A–E) Normal spatial learning and memory in Grin2b+/C456Y mice (P90–114) in the learning (A) and probe (B–E) phases of the initial (A–C) and reversal (A, D, E) sessions of the Morris water maze, as shown by time taken to escape to the platform, percent of time spent in target quadrant, and number of crossing over the former platform location. n = 12 mice for WT and HT, two-way ANOVA with Sidak’s test and Student t test. (F) Normal novel object–recognition memory in Grin2b+/C456Y mice (P70–80), as shown by the percent of time spent exploring a novel object relative to a familiar object. n = 12 mice for WT and HT, Student t test. The numerical data underlying this figure can be found in S3 Data. HT, heterozygous; ns, not significant; P, postnatal day; WT, wild type. (TIF) [file pbio.3000717.s006.tif]

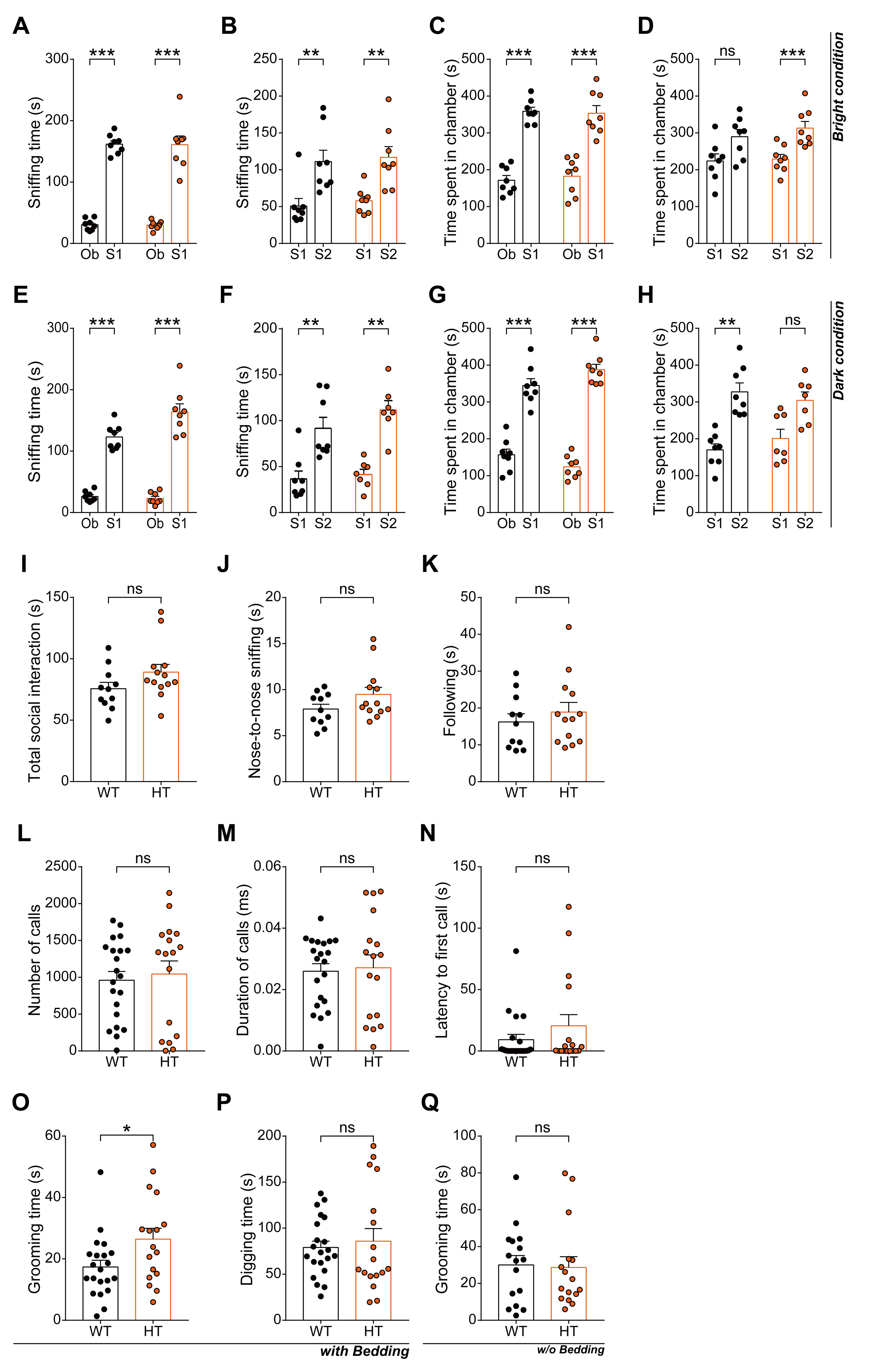

Supplement: S7 Fig — (A–H) Normal social approach and social novelty recognition in Grin2b+/C456Y mice (P79–80) in the three-chamber test under both bright-light and dark conditions, as shown by time spent sniffing the target and time spent in the chamber with the target. n = 8 mice for WT and HT (except for n = 7 for HT for social novelty recognition), **P < 0.01, ***P < 0.001, two-way ANOVA with Sidak’s test. (I–K) Normal social interaction in freely moving Grin2b+/C456Y mice (P62–89) in the direct social-interaction test, as shown by time spent in nose-to-nose sniffing or following and total time spent in social interaction (sniffing, following, and other social interactions). n = 11 mice for WT and 13 for HT, Student t test (except for Mann-Whitney test for following). (L–N) Normal courtship USVs in male Grin2b+/C456Y mice (P74–101) upon encountering a novel female mouse, as shown by the number of calls, duration of each call, and latency to the first call. n = 21 mice for WT and 17 for HT, Student t test (except for Mann-Whitney test for latency to first call). (O–P) Enhanced repetitive self-grooming (but normal digging) by Grin2b+/C456Y mice (P68–88) in home cages with bedding, but normal self-grooming in a novel chamber without bedding, as shown by time spent self-grooming (or digging). n = 21 mice for WT and 17 for HT for self-grooming in home cages with bedding, n = 16 mice for WT and HT for self-grooming in a novel chamber without bedding, *P < 0.05, Student t test. The numerical data underlying this figure can be found in S3 Data. HT, heterozygous; ns, not significant; P, postnatal day; USV, ultrasonic vocalization; WT, wild type. (TIF) [file pbio.3000717.s007.tif]

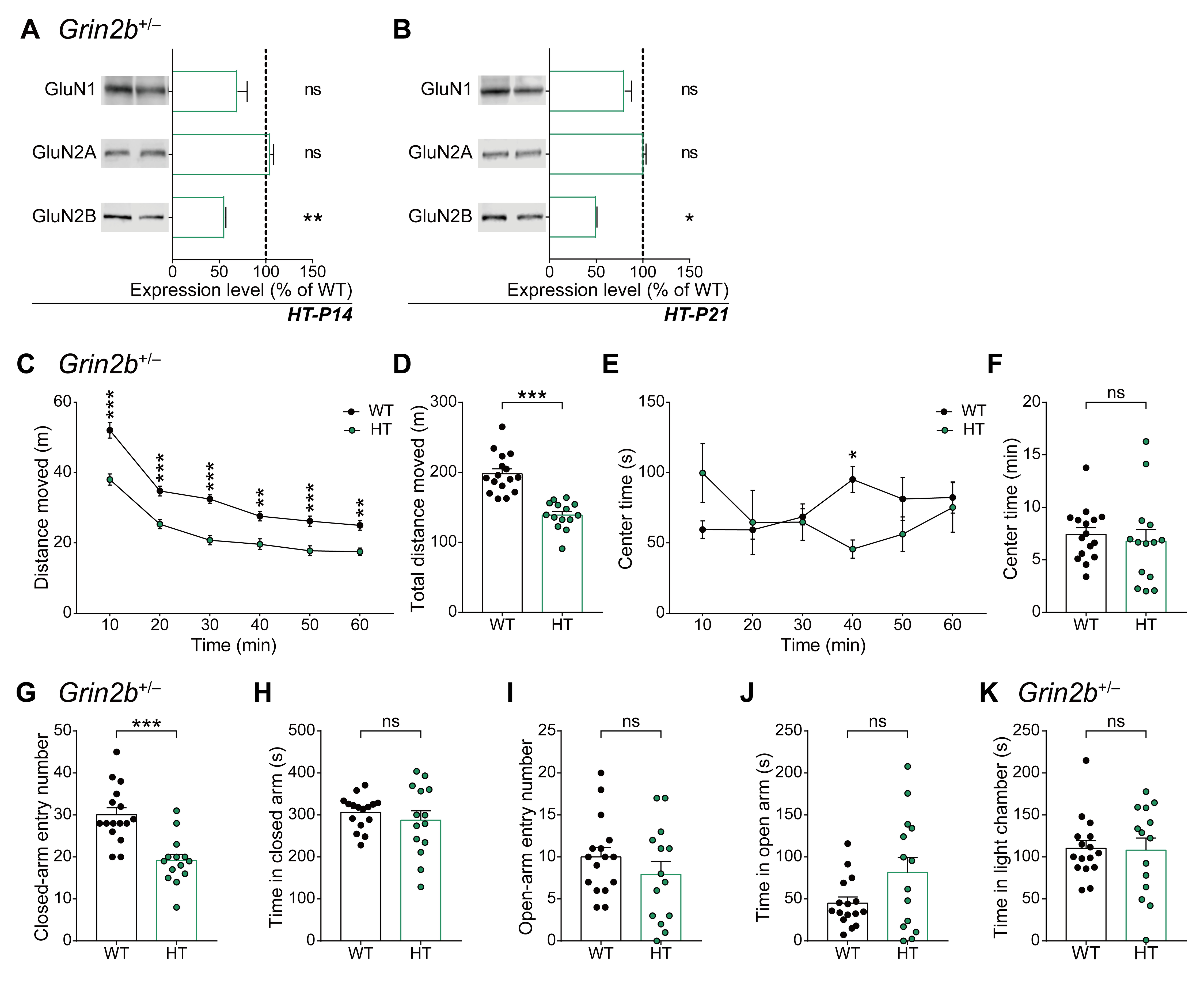

Supplement: S8 Fig — (A and B) Decreased levels of the GluN2B, but not GluN1 or GluN2A, protein in the Grin2b+/–mice brain. Whole-brain total lysates from Grin2b+/–mice at P14 and P21 were immunoblotted with anti-GluN1/2A/2B antibodies. For quantification, average levels of GluN1/2A/2B proteins from Grin2b+/–mice were normalized to those from WT mice. n = 4 mice for WT and HT, *P < 0.05, **P < 0.01, Student t test. (C–F) Hypoactivity and normal anxiety-like behavior in Grin2b+/–mice (P61–71) in the open-field test, as shown by distance moved and time spent in the center region of the open-field arena. n = 16 mice for WT and 14 for HT, *P < 0.05, **P < 0.01, ***P < 0.001, two-way ANOVA with Sidak’s test and Student t test. (G–J) Anxiolytic-like behavior in Grin2b+/–mice (P65–75) in the elevated plus-maze, as shown by the number of entries into and time spent in open/closed arms. n = 16 mice (WT) and 14 (HT), ***P < 0.001, Student t test. (K) Normal anxiety-like behavior in Grin2b+/–mice (P68–78) in the light-dark test, as shown by time spent in the light chamber. n = 16 mice (WT) and 14 (HT), Mann-Whitney test. The numerical data underlying this figure can be found in S3 Data. HT, heterozygous; ns, not significant; P, postnatal day; WT, wild type. (TIF) [file pbio.3000717.s008.tif]

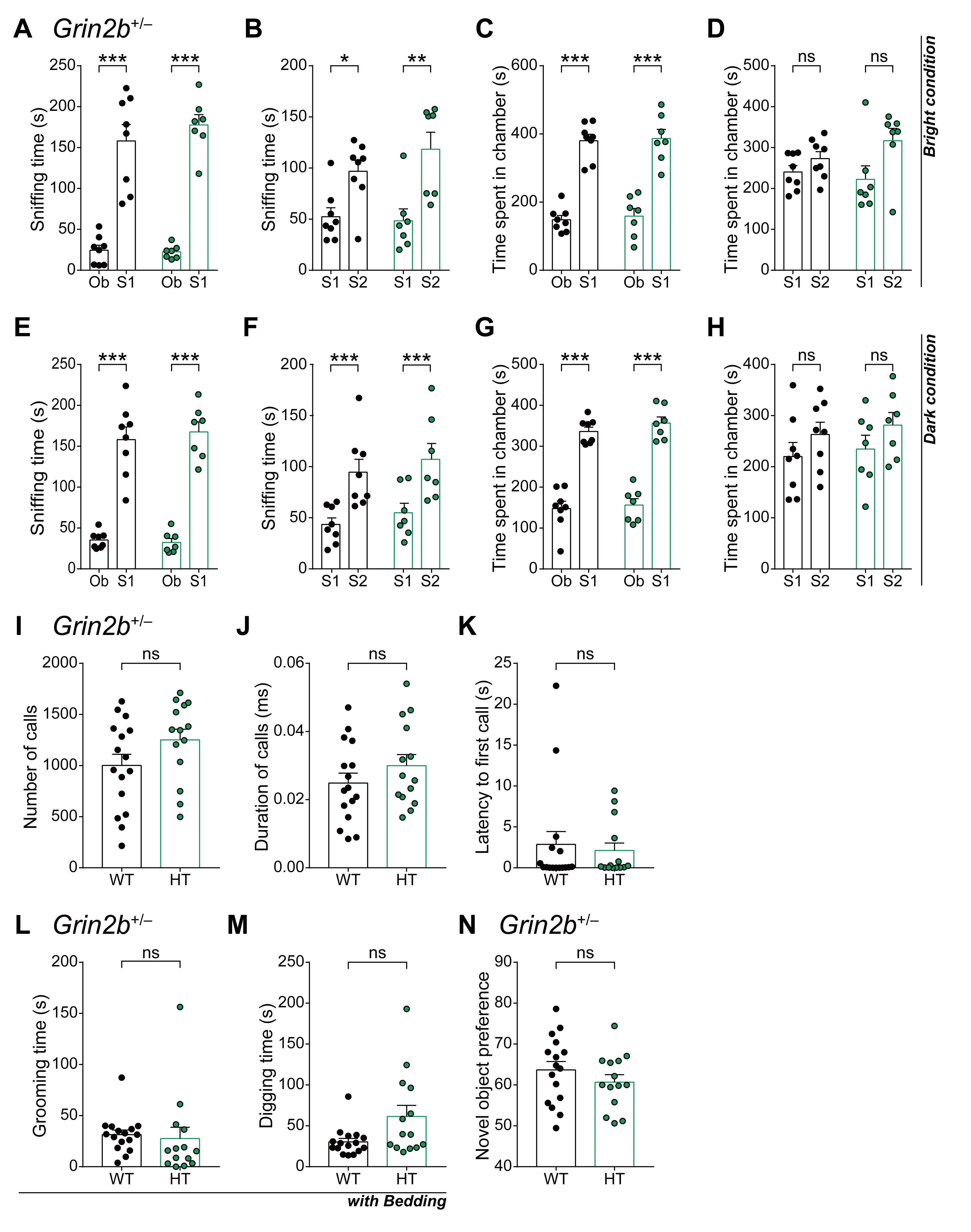

Supplement: S9 Fig — (A–H) Normal social approach and social novelty recognition in Grin2b+/–mice (P75–85) in the three-chamber test under both bright-light and dark conditions, as shown by time spent sniffing the target and time spent in the chamber with the target. n = 8 mice for WT and 7 for HT, *P < 0.05, **P < 0.01, ***P < 0.001, two-way ANOVA with Sidak’s test. (I–K) Normal courtship USVs in Grin2b+/–mice (P77–87) upon encountering a novel female mouse, as shown by the number of calls, duration of each call, and latency to the first call. n = 16 for WT and 14 for HT, Student t test except Mann-Whitney test for latency to first all. (L and M) Normal repetitive self-grooming and digging in Grin2b+/–mice (P77–87) in home cages with bedding, as shown by time spent self-grooming or digging. n = 16 (WT) and 14 (HT), Mann-Whitney test. (N) Normal novel object–recognition memory in Grin2b+/–mice (P63–73), as shown by the percent of time spent exploring a novel object relative to a familiar object. n = 16 (WT) and 14 (HT), Student t test. The numerical data underlying this figure can be found in S3 Data. HT, heterozygous; ns, not significant; P, postnatal day; USV, ultrasonic vocalization; WT, wild type. (TIF) [file pbio.3000717.s009.tif]

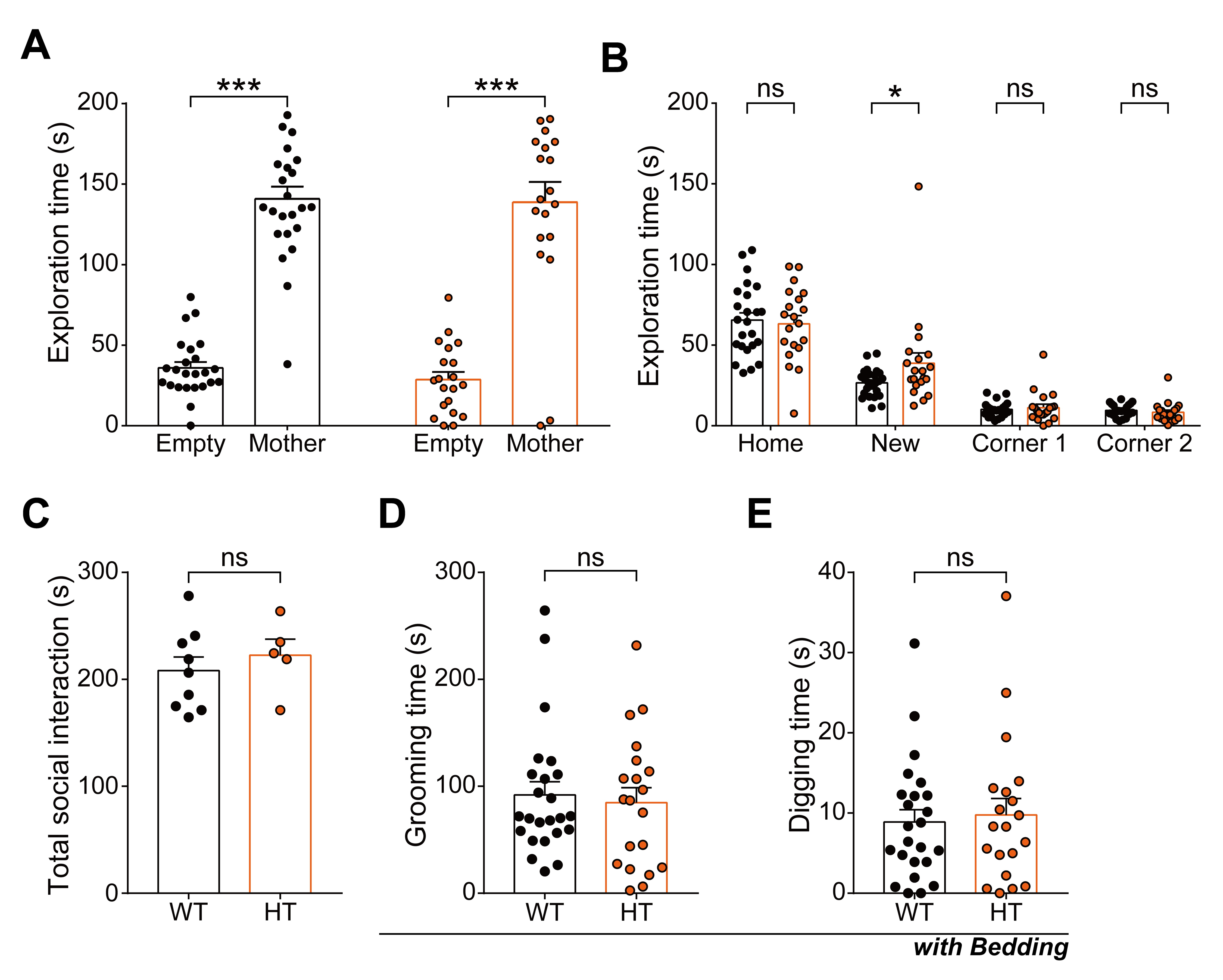

Supplement: S10 Fig — (A and B) Grin2b+/C456Y juveniles (P22–24) show normal behaviors in the maternal-homing test, as shown by the time spent with the reunited mother. Note that these mice showed normal exploration of the bedding materials from previous home cages (“Home”) suggestive of normal olfactory function; it is unclear why these mice prefer to explore the new corner (“New”). n = 24 mice for WT and 20 for HT, *P < 0.05, ***P < 0.001, two-way ANOVA with Sidak’s test. (C) Grin2b+/C456Y juveniles (P28–20) show normal social interaction in the juvenile play test, as shown by the total time spent in social interaction (nose-to-nose sniffing, following, and other social interactions). n = 9 pairs for WT and 5 pairs for HT, Student t test. (D and E) Grin2b+/C456Y juveniles (P24–26) show normal repetitive self-grooming and digging in home cages with bedding, as shown by time spent self-grooming or digging. n = 24 mice for WT and 20 for HT, Mann-Whitney test. The numerical data underlying this figure can be found in S3 Data. HT, heterozygous; ns, not significant; P, postnatal day; WT, wild type. (TIF) [file pbio.3000717.s010.tif]

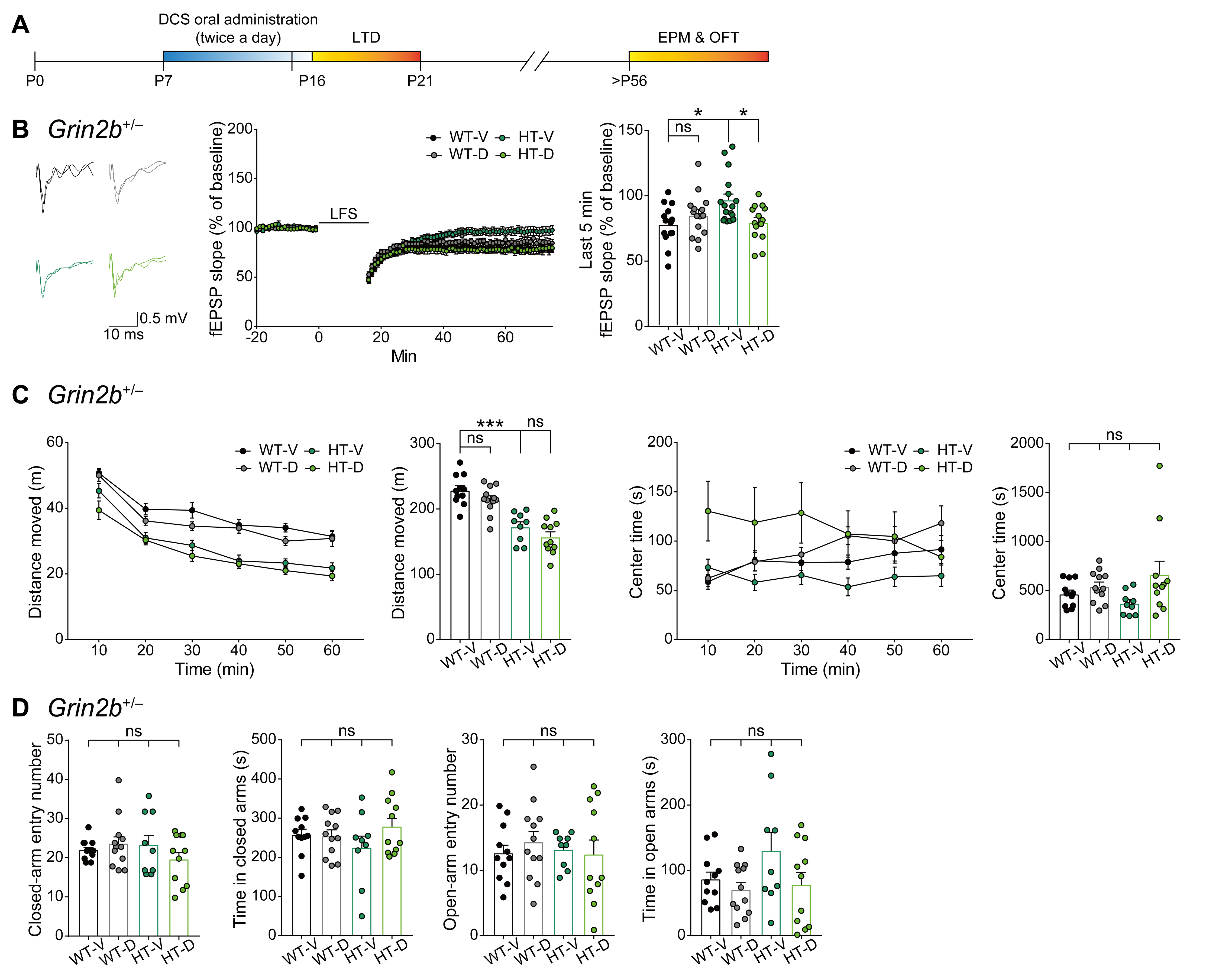

Supplement: S11 Fig — (A) Experimental strategy for chronic, oral DCS treatment (40 mg/kg), twice daily for 10 days (P7–16), in young Grin2b+/–mice followed by LTD measurements in juvenile mice (P17–21) and behavioral tests (OFT and EPM) in adult mice (>P56). (B) Early chronic DCS treatment normalizes LFS-LTD at SC-CA1 synapses in juvenile Grin2b+/–mice (P17–20). n = 14 mice (6) for WT_V (78.0% ± 4.1%), 15 (5) for WT_D (85.2% ± 4.2%), 17 (6) for HT_V (96.9% ± 4.5%), 14 (5) for HT_D (79.6% ± 3.7%), *P < 0.05, two-way ANOVA with Tukey’s test. (C) Early chronic DCS treatment has no effect on the hypoactivity in adult Grin2b+/–mice (P56–62). n = 11 mice for WT_D, 12 for WT_D, 9 for HT_V, 11 for HT_D, ***P < 0.001, two-way ANOVA with Tukey’s test. (D) Early chronic DCS treatment has no effect on the anxiety-like behavior in adult Grin2b+/–mice (P58–76), as shown by closed-arm time and open-arm entry/time. Note, however, that the early chronic drug treatment procedure (P7–16; twice a day for 10 days; oral) seems to modestly increase anxiety levels in the mutant mice, blunting the baseline difference in the closed-arm entry between WT-V and HT-V (see also panel G in S8 Fig), making it impossible to assess the effect of DCS on this value. n = 11 mice for WT_D, 12 for WT_D, 9 for HT_V, 11 for HT_D, two-way ANOVA with Tukey’s test. The numerical data underlying this figure can be found in S3 Data. DCS, D-cycloserine; EPM, elevated plus-maze; HT, heterozygous; HT_D, heterozygous with DCS; HT_V, heterozygous with vehicle; LFS, low-frequency stimulation; LTD, long-term depression; ns, not significant; OFT, open-field test; P, postnatal day; SC-CA1, Schaffer collateral-CA1 pyramidal; WT, wild type; WT_D, WT with DCS; WT_V, WT with vehicle. (TIF) [file pbio.3000717.s011.tif]

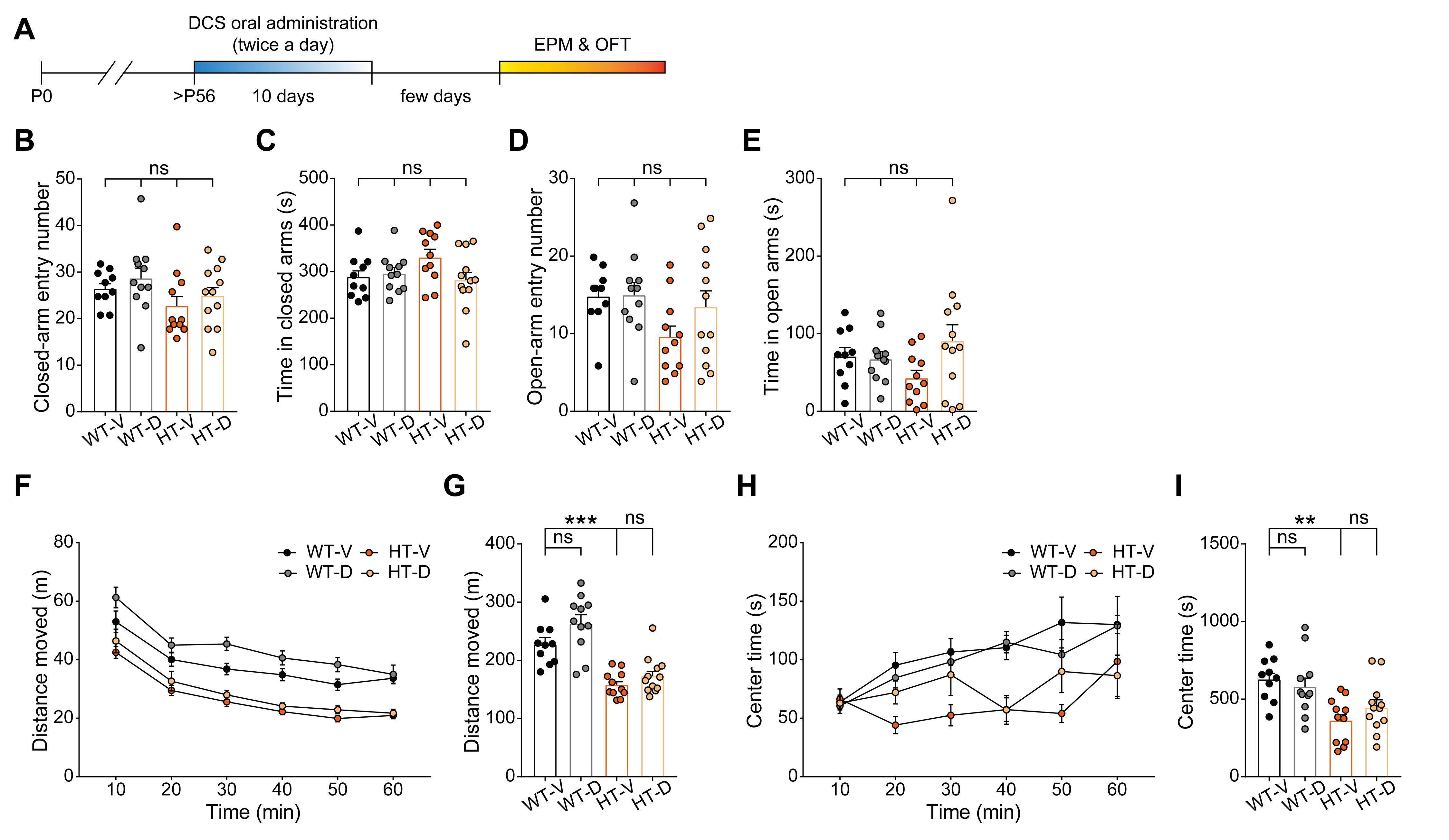

Supplement: S12 Fig — (A–E) Late chronic oral DCS treatment (40 mg/kg; twice daily for 10 days) in adult Grin2b+/C456Y mice (P57–66) does not affect anxiolytic-like behavior in the EPM. Note, however, that the chronic drug treatment procedure (oral drug administration twice a day for 10 days using a restrainer, unlike the pup situation in which pups were gently grabbed) seems to substantially increase anxiety levels in the mutant mice, blunting the baseline difference in EPM variables between WT-V and HT-V (see also Fig 3E–3H and Fig 4F–4I), making it impossible to assess the effect of DCS on these values. n = 10 mice for WT_D, 11 for WT_D, 11 for HT_V, 12 for HT_D, two-way ANOVA with Tukey’s test. (F–I) Late chronic oral DCS treatment (40 mg/kg; twice daily for 10 days) in adult Grin2b+/C456Y mice (P57–66) does not affect hypoactivity in the OFT. n = 10 mice for WT_D, 11 for WT_D, 11 for HT_V, 12 for HT_D, **P < 0.01, ***P < 0.001, two-way ANOVA with Tukey’s test. The numerical data underlying this figure can be found in S3 Data. DCS, D-cycloserine; EPM, elevated plus-maze; HT, heterozygous; HT_D, heterozygous with DCS; HT_V, heterozygous with vehicle; ns, not significant; OFT, open-field test; P, postnatal day; WT, wild type; WT_D, WT with DCS; WT_V, WT with vehicle. (TIF) [file pbio.3000717.s012.tif]
