# Supplementary material for: Early correction of synaptic long-term depression improves abnormal anxiety-like behavior in adult GluN2B-C456Y-mutant mice
Source: PLoS Biol. 2020 Apr 30;18(4):e3000717. doi: 10.1371/journal.pbio.3000717 (PMC7217483; doi:10.1371/journal.pbio.3000717)
Supplement: S2 Data — (DOCX) [file pbio.3000717.s014.docx]

**Early correction of synaptic long-term depression improves abnormal anxiety-like behavior in adult GluN2B-C456Y-mutant mice**

Wangyong Shin ^1,#^, Kyungdeok Kim ^1,#^, Benjamin Serraz ^2^, Yi Sul Cho ^3^, Doyoun Kim ^4^, Muwon Kang ^1^, Eun-Jae Lee ^5^, Hyejin Lee ^1^, Yong Chul Bae ^3^, Pierre Paoletti ^2^, and Eunjoon Kim ^1,4,*^

^1^Department of Biological Sciences, Korea Advanced Institute of Science and Technology (KAIST), Daejeon, Korea; ^2^Institut de Biologie de l'Ecole Normale Supérieure (IBENS), Ecole Normale Supérieure, Université PSL, CNRS, INSERM, F-75005, Paris, France; ^3^​Department of Anatomy and Neurobiology, School of Dentistry, Kyungpook National University, Daegu, Korea; ^4^Center for Synaptic Brain Dysfunctions, Institute for Basic Science (IBS), Daejeon, Korea; ^5^Department of Neurology, Asan Medical Center, University of Ulsan, College of Medicine, Seoul, Korea; ^#^These authors contributed equally to the study; *Corresponding author (kime@kaist.ac.kr).

**SUPPLEMENTARY METHODS**

**Animals**

To discriminate *Grin2b^+/+^*, *Grin2b^+/C456Y^* and *Grin2b^C456Y/C456Y^* mice, following PCR primer sets were used. Forward1: ACGACTCTTTGTGGAGGAGGG, Reverse1: CCATATCACAGCTTACTTCAATGT. *Grin2b^+/–^* mice were purchased from RIKEN. To discriminate *Grin2b^+/+^* mice and *Grin2b^+/–^* mice, following PCR primer sets were used. Forward1: AGAGTCGACGAGCTGAAGATGAAGCCCAGC, Reverse1: GCCTGCTTGCCGAATATCATGGTGGAAAAT, Reverse2: CGGGGAACTACTGAGAGATGATGGAAGTCA. Young mice were weaned at postnatal days 21–27. After weaning, a maximum of 8 littermates of mixed genotype was group-housed before experiments. Animals were housed under 12 hours (13:00-01:00) dark/light cycle environment, and were fed *ad libitum*. All animals were bred and maintained according to the Requirements of Animal Research at KAIST and all procedures were approved by the Committees of Animal Research at KAIST (KA2016-31).

**Molecular modeling**

The structure of the mouse diheteromeric complex of NMDARs, composed by two GluN1 and two GluN2B subunits, was modeled by using a homology modelling server (SWISS-MODEL) based on the crystal structure of the rat NMDAR complex (PDB ID: 4PE5) as an initial model [1]. The structure of the GluN2B subunit harboring missense mutation (p.C456Y) was modeled using mutagenesis function in PyMOL software (version 1.3). Energy minimization and loop flexible modeling were performed using Modeller software [2]. The interaction residues involved in inter-domain interaction between the ATD and LBD of GluN2B were calculated by protein interaction calculator (PIC) server [3]. All structural figures were prepared using PyMOL software (version 1.3).

## DNA sequencing

DNA region containing *Grin2b* exon 6 was amplified by PCR using the following primer sets. Forward: GGCTTATTCTCTTTCCTCCTT, Reverse: ATGAGGCAGCACATTACTGG. Amplified DNA was subjected to DNA sequencing to confirm nucleotide replacement.

## Brain homogenates and immunoblot

Brain homogenates from *Grin2b^+/+^*, *Grin2b^+/C456Y^*, *Grin2b^C456Y/C456Y^* and *Grin2b^+/–^* mice were prepared as described [4]. Briefly, mouse brains at different developmental stages (E20, P14, P21, P28 and P56 for KI mice, P14 and P21 for conventional mice) were homogenized in ice-cold homogenization buffer (0.32 M sucrose, 10 mM HEPES, pH 7.4, 2 mM EDTA, protease inhibitors and phosphatase inhibitors). The homogenates were centrifuged at 1100 x g for 10 min. The resulting supernatant was centrifuged again at 12,000 x g for 15 min. The pellet was resuspended in homogenization buffer and centrifuged at 13,000 x g for 15 min (the resulting pellet is P2 or crude synaptosomes). This sample was immunoblotted with antibodies for GluN1 (BD, 556308, 1:1,000), GluN2A (Millipore, 07-632, 1:1,000) and GluN2B (Neuromab, 75-101, 1:1,000).

**Electron microscopy**

Wild type and *Grin2b^+/C456Y^* mice were deeply anesthetized with a mixture of ketamine (120 mg/kg) and xylazine (10mg/kg) and were intracardially perfused with 10 ml of heparinized normal saline, followed by 50 ml of a freshly prepared fixative of 2.5% glutaraldehyde and 1% paraformaldehyde in 0.1 m phosphate buffer (PB, pH 7.4). The hippocampus was removed from the whole brain, postfixed in the same fixative for 2 hours and stored in PB (0.1 M, pH 7.4) overnight at 4 °C. Sections (70 μm) were cut transversely on a Vibratome. The sections were osmicated with 1% osmium tetroxide (in 0.1 mPB) for 1 hour, dehydrated in graded alcohols, flat embedded in Durcupan ACM (Fluka), and cured for 48 hrs at 60 °C. Small pieces containing stratum radiatum of the hippocampal CA1 region were cut out of the wafers and glued onto the plastic block by cyanoacrylate. Ultrathin sections were cut and mounted on Formvar-coated single-slot grids, stained with uranyl acetate and lead citrate, and examined with an electron microscope (Hitachi H-7500; Hitachi) at 80 kV accelerating voltage. Thirty-two micrographs representing 491.9 μm^2^ neuropil regions in each mouse were photomicrographed at a 40,000× magnification and used for quantification. The density of postsynaptic densities (PSD density), PSD length, PSD thickness, and the proportion of perforated PSDs were quantified. The measurements were all performed by an experimenter blind to the genotype. Digital images were captured with GATAN DigitalMicrograph software driving a CCD camera (SC1000 Orius; Gatan) and saved as TIFF files. The brightness and contrast of the images were adjusted using Adobe Photoshop 7.0 (Adobe Systems).

**Experiments on recombinant GluN1/GluN2B receptors**

***Molecular biology***

The pcDNA3-based plasmids for rodent NMDAR subunits (rat GluN1-1a, named GluN1 herein and mouse GluN2B), the site-directed mutagenesis and sequencing procedures have been previously described [5].

***Electrophysiology***

Oocytes from female *Xenopus laevis* were prepared and voltage-clamped as described previously [6]. Oocyte harvest was performed in accordance with the European directives 2010/63/EU on the Protection of Animals used for Scientific Purposes. Recombinant NMDA receptors were expressed in oocyte after coinjection of 36.8 nL of a mixture of cDNAs, each at the concentration of 30 ng/µL (nuclear injection), coding for wild-type GluN1 and wild-type or mutated GluN2B subunits (ratio 1:1). Data were collected using pClamp10 and analyzed using Kaleidagraph4 and SigmaPlot11. The standard external solution contained (in mM): 100 NaCl, 2.5 KCl, 0.3 BaCl_2_, 5 HEPES, 0.01 DTPA (diethylenetriamine-pentaacetic acid), pH 7.3. For pH and spermine experiments, an HEPES-enriched external medium was used [5, 7]. To maximize spermine potentiating effects, spermine experiments were performed at an external pH of 6.5, as previously described [5]. For zinc experiments, DTPA was omitted. NMDAR-mediated currents were induced by co-application of saturating concentrations of L-glutamate and glycine (100 µM each), except for experiments with D-cycloserine where glycine was omitted and replaced by 100 µM D-cycloserine. Recordings were performed at a holding potential of -60 mV and at room temperature.

***Pharmacology and data analysis***

Expression level was determined based on NMDAR-mediated currents elicited by saturating concentration of agonists, two or three days following oocyte nuclear injection (for wild-type and mutated GluN2B containing NMDAR respectively). Glutamate and glycine dose-response curve (DRC) experiments were performed in the presence of 100 µM of the respective co-agonist. Agonist DRCs were fitted with the following Hill equation: I_rel_ = 1/(1+(EC_50_/[A])^nH^), with EC_50_ and nH as free parameters. Determination of MK-801 inhibition time constant (τ_on_) was performed and analyzed as in Mony et al. [5]. MK-801 was applied at 10 nM. Proton DRCs were analyzed and fitted as in Gielen et al. [7], with pHIC_50_ and nH as free parameters. Zinc DRCs were fitted with the following equation: I_rel_ = 1-1/(1+(IC_50_/[C])^nH^), with IC_50_ and nH as free parameters, and C the concentration of free zinc corrected for an estimated contaminant zinc concentration of 100 nM [8]. Spermine experiments were analyzed as described in Mony et al. [5].

**Quantitative PCR**

cDNAs was synthesized using TOPscript^TM^ Cdna synthesis kit (Enzynomics, EZ005). qPCR was performed using SsoAdvanced^TM^SYBR® Green Supermix (BIORAD, 170-8882AP), CFX96^TM^Real-Time system. The following primer sets were used to target indicated exons. Grin2b Exon 3 Forward: GATTCTGCATTGTGAGCCGC, Grin2b Exon 3 Reverse: TAAGGGAGAGCGCATGTTGG, Grin2b Exon 4 Forward: ACGAGCTGAAGATGAAGCCC, Grin2b Exon 4 Reverse: CCCGGGGAACTACTGAGAGA, Grin2b Exon 11 Forward: CCCATCTTTCACCATCGGCA, Grin2b Exon 11 Reverse: AGCTGGCCAGGAAAATGACA, Grin2b Exon 14 Forward: GACTTCTCACCCCCTTTCCG, Grin2b Exon 14 Reverse: TTCAGGGAGAGCAAGGCATC, Grin1 Exon 3 Forward: AGCATCCACCTGAGCTTCCT, Grin1 Exon 3 Forward Reverse: CTCCTCCAGCAACGTCTCCA, Grin1 Exon 7 Forward: CGGACTTCAGCTAATCAACGG, Grin1 Exon 7 Reverse: TGAACAGTGGTCCTGTCTTCC, Grin1 Exon 12 Forward: AGCAACAAAAAGGAGTGGAACG, Grin1 Exon 12 Reverse: CCTGGTACTTGAAGGGCTTGG.

## Brain slices for electrophysiology

For hippocampal electrophysiological experiments, acute sagittal brain slices (300 μm thickness for whole-cell patch and 400 μm for field recording) of *Grin2b*^+/+^ and *Grin2b^+/C456Y^* were obtained using a vibratome (Leica VT1200) after anesthetizing animals with isoflurane (Terrell). Brains were extracted and sliced in ice-cold dissection buffer containing (in mM) 212 sucrose, 25 NaHCO_3_, 5 KCl, 1.25 NaH_2_PO_4_, 0.5 CaCl_2_, 3.5 MgSO_4_, 10 D-glucose, 1.25 L-ascorbic acid and 2 Na-pyruvate bubbled with 95% O_2_/5% CO_2_. The slices were transferred to 32 °C recovery chamber with normal ACSF (in mM: 125 NaCl, 2.5 KCl, 1.25 NaH_2_PO_4_, 25 NaHCO_3_, 10 glucose, 2.5 CaCl_2_ and 1.3 MgCl_2_ oxygenated with 95% O_2_/5% CO_2_). After 30 min recovery in 32 °C, slices were recovered for an additional 30 min at 20-25 °C. For the recording, a single slice was transferred to a submerged-type chamber at 27–28 °C with circulating ACSF (2 ml/min) saturated with 95% O_2_/5% CO_2_. Stimulation and recording pipettes were pulled from thin-walled borosilicate glass capillaries (30-0065, Harvard Apparatus) with resistance 2.5–3.5 MΩ using a micropipette electrode puller (PC-10, Narishige).

## Whole-cell patch

Whole-cell patch-clamp recordings of hippocampal CA1 pyramidal neurons were made using a MultiClamp 700B amplifier (Molecular Devices) and Digidata 1550 (Molecular Devices). During whole-cell patch-clamp recordings, series resistance was monitored each sweep by measuring the peak amplitude of the capacitance currents in response to short hyperpolarizing step pulse (5 mV, 40 ms); only cells with a change in < 20% were included in the analysis. To measure the excitability of hippocampal CA1 cells, recording pipettes (2.5–3.5 MΩ) were filled with an internal solution containing the following (in mM): 137 K-gluconate, 5 KCl, 10 HEPES, 0.2 EGTA, 10 Na-phosphocreatine, 4 Mg-ATP, and 0.5 Na-GTP, with pH 7.2, 280 mOsm. To inhibit postsynaptic responses, picrotoxin (100 μM), NBQX (10 μM) and D-AP5 (50 μM) were added. After rupturing the cell, the current was clamped and RMP was measured. Cells with RMP larger than -60 mV were not used. After stabilizing the cell, RMP was adjusted by -65 mV. The current input was increased from 0 to 360 pA in increments of 30 pA per sweep. Each current was injected with a time interval of 15 seconds. To measure mEPSCs in hippocampal CA1 pyramidal neurons, recording pipettes (2.5–3.5 MΩ) were filled with an internal solution containing the following (in mM): 100 CsMeSO_4_, 10 TEA-Cl, 8 NaCl, 10 HEPES, 5 QX-314-Cl, 2 Mg-ATP, 0.3 Na-GTP and 10 EGTA, with pH 7.25, 295 mOsm. Whole-cell recording of mEPSCs was made in neurons at a holding potential of -70 mV. TTX (1 μM) and picrotoxin (100 μM) were added to ACSF to inhibit spontaneous action potential-mediated synaptic currents and IPSCs, respectively. To record sEPSCs, picrotoxin (100 mΜ) but not TTX was added to ACSF. To measure mIPSCs in hippocampal CA1 pyramidal neurons, recording pipettes (2.5–3.5 MΩ) were filled with an internal solution containing the following (in mM): 120 CsCl, 10 TEA-Cl, 8 NaCl, 10 HEPES, 5 QX-314-Cl, 4 Mg-ATP, 0.3 Na-GTP and 10 EGTA, with pH 7.35, 280 mOsm. TTX (1 μM), NBQX (10 μM) and D-AP5 (50 μM) were added to ACSF to inhibit spontaneous action potential-mediated synaptic currents, AMPAR-mediated currents and NMDAR-mediated currents, respectively. To record sIPSCs, NBQX (10 μM) and D-AP5 (50 μM) but not TTX were added to ACSF. For measuring NMDAR/AMPAR ratio, CA1 pyramidal neurons were voltage clamped at -70 mV, and EPSCs were evoked at every 15 s. AMPAR-mediated EPSCs were recorded at -70 mV, and 20 consecutive responses were recorded after stable baseline. After recording AMPAR-mediated EPSCs, holding potential was changed to +40 mV to record NMDAR-mediated EPSCs. The NMDA component at 60 ms after the stimulation was used for analysis. The NMDA/AMPA ratio was determined by dividing the mean value of 20 NMDA components of EPSCs by the mean value of 20 AMPAR-mediated EPSC peak amplitudes. To measure evoked IPSC/EPSC, recording pipettes (2.5–3.5 MΩ) were filled with an internal solution containing the following (in mM): 120 CsMeSO_4_ 15 CsCl, 10 TEA-Cl, 8 NaCl, 10 HEPES, 5 QX-314-Cl, 4 Mg-ATP, 0.3 Na-GTP and 0.25 EGTA, with pH 7.35, 280 mOsm. D-AP5 (50 μM) were added to ACSF to inhibit NMDAR-mediated currents. CA1 pyramidal neurons were voltage clamped at -70 mV, and EPSCs were evoked at every 15 s. AMPAR-mediated EPSCs were recorded at -70 mV, and 20 consecutive responses were recorded after stable baseline. After recording AMPAR-mediated EPSCs, holding potential was changed to 0 mV to record GABAR-mediated IPSCs. The IPSC/EPSC ratio was determined by dividing the mean value of 20 GABAR components of IPSCs by the mean value of 20 AMPAR-mediated EPSC peak amplitudes. Data were acquired by Clampex 10.2 (Molecular Devices) and analyzed by Clampfit 10 (Molecular Devices). Drugs were purchased from Abcam (TTX), Tocris (NBQX, D-AP5) and Sigma (picrotoxin).

## Field recording

For field recordings, fEPSPs were recorded in the stratum radiatum of the hippocampal CA1 region using pipettes filled with ACSF. fEPSP was amplified (Multiclamp 700B, Molecular Devices) and digitized (Digidata 1550, Molecular Devices) for measurements. The Schaffer collateral pathway was stimulated, and baseline responses were collected every 20 s with a stimulation intensity that yielded a half-maximal response. For input/output experiment, after acquiring a stable baseline, a series of increasing input stimuli were given to evoke output signals. Obtained fEPSP slopes and fiber volleys were then interpolated by linear fits to plot input/output relationships. For paired-pulse ratio experiments, stimuli with indicated inter-pulse intervals (25, 50, 75, 100, 200, 300 ms) were given, and pairs of peak amplitudes were recorded and analyzed to calculate the ratio of the amplitudes. To induce LTD, low-frequency stimulation (1 Hz, 15 min) or DHPG (50 μM, 10 min; for mGluR-LTD) was applied. To induce LTP, high-frequency stimulation (100 Hz, 1 s) or theta-burst stimulation (10 trains of 4 pulses at 100 Hz) was applied. Data were acquired by Clampex 10.2 (Molecular Devices) and analyzed by Clampfit 10 (Molecular Devices).

## Animal behavioral tests

All behavioral assays were performed using littermates or age-matched animals during light-off periods in their home cages, except for the automated 48-hr movement analysis with the LABORAS apparatus. All behavioral test results were analyzed in a blind manner.

## Open field test

Mice were placed in an open field box (40 x 40 x 40 cm) and recorded for 60 min (20 min for juvenile open field test). The center-zone line was drawn at the place 10 cm apart from the edge. The testing room was illuminated at 0 lux. Mice movements were analyzed using EthoVision XT 10 program (Noldus).

## Elevated plus-maze test

The elevated plus-maze test was performed as described [9]. The elevated plus-maze consisted of two open arms, two closed arms, and a center zone, and was elevated to a height of 50 cm above the floor. Mice were placed in the center zone in the beginning of the test and allowed to explore the space for 8 min. The data was analyzed using EthoVision XT 10 program (Noldus).

## Light-dark chamber test

The apparatus for the light-dark test consisted of light (~400 lux) and dark (~0 lux) chambers adhered to each other. The size of the light chamber was 20 x 30 x 20 cm, and that of the dark chamber was 20 x 13 x 20 cm. An entrance enabled mice to freely move across the light and dark chambers. Mice were introduced to the center of the light chamber and allowed to explore the apparatus freely for 10 min. The time spent in light and dark chambers were measured using EthoVision XT 10 program (Noldus).

## Morris water maze test

Mice were trained to find the hidden platform (10 cm diameter) in a white plastic tank (120 cm diameter). Mice were given 3 trials per day with an inter-trial interval of 30 min. The learning-phase experiments of the water maze were performed for eight consecutive days, followed by the probe test on day 9 where mice were given 1 min to find the removed platform. For reversal training (days 10–15), the location of the platform was switched to the opposite position from the previously trained one, and mice were trained to learn the new position of the platform. Target quadrant occupancy and the exact number of crossings over the former platform location during the probe test were measured using EthoVision 10 program (Noldus).

## Novel object recognition test

The object recognition test was performed in the open-field box. On the first day, mice were allowed to explore two identical objects for 10 min. 24 hours later, mice were placed the same box where one of the two objects was replaced with a new one. Exploration time for each object was measured.

## Three-chamber social interaction test

The three-chamber test, designed to measure social approach and social novelty recognition in rodents [10-12], was performed as described previously [13, 14]. Briefly, a subject mouse was placed in a three-chamber apparatus, which has 3 chambers (one center and two side chambers). In the first session, a mouse could freely move around the whole three chambers with two empty containers in the left or right corner for 10 min. The mouse was then gently guided to the center chamber while a novel ‘Object’ and a wild-type stranger mouse ‘Stranger 1 (129Sv strain)’ were placed in the two containers. The subject mouse was then allowed to freely explore all three chambers for 10 min. In the third session, the subject mouse was again gently guided to the center chamber while the ‘Object’ was replaced with a wild-type ‘Stranger 2’ mouse (129Sv strain). The subject mouse again was allowed to freely explore all three chambers for 10 min.

## Direct interaction test and juvenile play test

Each mouse was habituated in a direct social interaction box for 30 min on the day before the experiment. On test day, pairs of mice with the same age and genotype, which have not met before, were placed in a direct interaction box, and their interactions were recorded for 10 min. For the juvenile play test, subject mice were habituated in a new home cage with bedding for 1 hour, after isolation from their mothers and siblings, on test day. Pairs of mice in the same age, sex, and genotype that have not met before were placed in a new home cage with bedding, and their interactions were recorded for 10 min. Nose-to-nose sniffing, following, mounting and allo-grooming were quantified manually and used to calculate total social interaction.

## Ultrasonic vocalization

An ultrasound microphone (Avisoft) and Avisoft Recorder software were used to record mouse ultrasonic vocalizations (USVs), a form of social communication in rodents [15]. For recording USVs, a subject male mouse was placed in a home cage with an age-matched unfamiliar C57BL/6J female mouse, and USVs were recorded for 5 min. For pup USVs, pups at the age of postnatal days 4, 6, 8, 10 and 12 were separated from dams and placed in a glass container, and USVs were recorded for 3 min. Recorded USVs were analyzed as previously described [16]. Briefly, Avisoft SASLab Pro software (RRID:SCR_014438) was used to analyzed USVs. Signals were filtered from 1 Hz to 100 kHz and digitized with a sampling frequency of 250 kHz, 16 bits per sample (Avisoft UltraSoundGate 116H). To generate spectrograms, the following parameters were used: FFT length: 256, frame size: 100, window: FlatTop, overlap: 75%, resulting in a frequency resolution of 977 Hz and a temporal resolution of 0.256 ms. Frequencies lower than 45 kHz were filtered out to reduce background white noises.

**Repetitive behaviors**

For repetitive behavioral tests, a subject mouse was placed in a novel home cage with or without bedding, and their behaviors were recorded for 20 min. For juvenile repetitive behaviors, subject mice were placed in a novel home cage with bedding, and their behaviors were recorded for 20 min. Self-grooming and digging behaviors from the last 10 min were quantified manually.

**Maternal homing test**

Juvenile mice were separated from their mothers for at least 30 min before testing. The testing consists of a nest homing phase followed by a maternal homing phase. For the nest homing phase, bedding materials from the original home cage (Home) and fresh bedding (New) were placed in the opposite corners of an open-field box. Subject mice were placed in one empty corner, and their behaviors were recorded for 3 min. For the maternal homing phase, an empty container and the container with the mother of the subject mouse were placed in the two opposite empty corners of the box after finishing 3 min nest homing phase. Subject mice were placed in the corner with bedding from the home cage, and their behaviors were recorded for 5 min. Time spent with bedding and time spent sniffing the containers were quantified manually.

**D-cycloserine administration**

For bath application of D-cycloserine for brain slices, D-cycloserine (DCS; Sigma, C6880; 10 μΜ) was added to ACSF. Hippocampal slices were exposed to ACSF with DCS at least 30 minutes before recording. For vehicle treatment, the same volume of saline was added to ACSF. For chronic and oral administration of DCS to young mice, DCS was dissolved in 0.1% saccharin-based drinking water (80 mg/ml) and orally administered to mice (40 mg/kg) twice a day during postnatal days 7–16. For chronic and oral administration of DCS to adult mice, tube restrainer was used to restrict unexpected movement of subject mice. For acute DCS injection in adult mice, subject mice received an intraperitoneal injection of DCS (20 mg/kg), or the same volume of saline, 30 min before behavioral tests.

**References**

1. Waterhouse A, Bertoni M, Bienert S, Studer G, Tauriello G, Gumienny R, et al. SWISS-MODEL: homology modelling of protein structures and complexes. Nucleic acids research. 2018;46(W1):W296-W303. doi: 10.1093/nar/gky427. PubMed PMID: 29788355; PubMed Central PMCID: PMCPMC6030848.

2. Fiser A, Do RK, Sali A. Modeling of loops in protein structures. Protein Sci. 2000;9(9):1753-73. doi: 10.1110/ps.9.9.1753. PubMed PMID: 11045621; PubMed Central PMCID: PMCPMC2144714.

3. Tina KG, Bhadra R, Srinivasan N. PIC: Protein Interactions Calculator. Nucleic acids research. 2007;35(Web Server issue):W473-6. doi: 10.1093/nar/gkm423. PubMed PMID: 17584791; PubMed Central PMCID: PMCPMC1933215.

4. Lee EJ, Lee H, Huang TN, Chung C, Shin W, Kim K, et al. Trans-synaptic zinc mobilization improves social interaction in two mouse models of autism through NMDAR activation. Nat Commun. 2015;6:7168. Epub 2015/05/20. doi: 10.1038/ncomms8168. PubMed PMID: 25981743; PubMed Central PMCID: PMCPMC4479043.

5. Mony L, Zhu S, Carvalho S, Paoletti P. Molecular basis of positive allosteric modulation of GluN2B NMDA receptors by polyamines. The EMBO journal. 2011;30(15):3134-46. doi: 10.1038/emboj.2011.203. PubMed PMID: 21685875; PubMed Central PMCID: PMC3160180.

6. Paoletti P, Ascher P, Neyton J. High-affinity zinc inhibition of NMDA NR1-NR2A receptors. The Journal of neuroscience : the official journal of the Society for Neuroscience. 1997;17(15):5711-25. PubMed PMID: 9221770.

7. Gielen M, Le Goff A, Stroebel D, Johnson JW, Neyton J, Paoletti P. Structural rearrangements of NR1/NR2A NMDA receptors during allosteric inhibition. Neuron. 2008;57(1):80-93. doi: 10.1016/j.neuron.2007.11.021. PubMed PMID: 18184566; PubMed Central PMCID: PMC2679256.

8. Rachline J, Perin-Dureau F, Le Goff A, Neyton J, Paoletti P. The micromolar zinc-binding domain on the NMDA receptor subunit NR2B. The Journal of neuroscience : the official journal of the Society for Neuroscience. 2005;25(2):308-17. doi: 10.1523/JNEUROSCI.3967-04.2005. PubMed PMID: 15647474.

9. Walf AA, Frye CA. The use of the elevated plus maze as an assay of anxiety-related behavior in rodents. Nature protocols. 2007;2(2):322-8. doi: 10.1038/nprot.2007.44. PubMed PMID: 17406592; PubMed Central PMCID: PMCPMC3623971.

10. Silverman JL, Yang M, Lord C, Crawley JN. Behavioural phenotyping assays for mouse models of autism. Nature reviews Neuroscience. 2010;11(7):490-502. doi: 10.1038/nrn2851. PubMed PMID: 20559336; PubMed Central PMCID: PMC3087436.

11. Moy SS, Nadler JJ, Young NB, Nonneman RJ, Grossman AW, Murphy DL, et al. Social approach in genetically engineered mouse lines relevant to autism. Genes, brain, and behavior. 2009;8(2):129-42. doi: 10.1111/j.1601-183X.2008.00452.x. PubMed PMID: 19016890; PubMed Central PMCID: PMC2659808.

12. Crawley JN. Designing mouse behavioral tasks relevant to autistic-like behaviors. Mental retardation and developmental disabilities research reviews. 2004;10(4):248-58. doi: 10.1002/mrdd.20039. PubMed PMID: 15666335.

13. Won H, Lee HR, Gee HY, Mah W, Kim JI, Lee J, et al. Autistic-like social behaviour in Shank2-mutant mice improved by restoring NMDA receptor function. Nature. 2012;486(7402):261-5. Epub 2012/06/16. doi: 10.1038/nature11208. PubMed PMID: 22699620.

14. Chung W, Choi SY, Lee E, Park H, Kang J, Park H, et al. Social deficits in IRSp53 mutant mice improved by NMDAR and mGluR5 suppression. Nat Neurosci. 2015;18(3):435-43. Epub 2015/01/27. doi: 10.1038/nn.3927. PubMed PMID: 25622145.

15. Scattoni ML, Crawley J, Ricceri L. Ultrasonic vocalizations: a tool for behavioural phenotyping of mouse models of neurodevelopmental disorders. Neuroscience and biobehavioral reviews. 2009;33(4):508-15. doi: 10.1016/j.neubiorev.2008.08.003. PubMed PMID: 18771687; PubMed Central PMCID: PMCPMC2688771.

16. Kim R, Kim J, Chung C, Ha S, Lee S, Lee E, et al. Cell-Type-Specific Shank2 Deletion in Mice Leads to Differential Synaptic and Behavioral Phenotypes. J Neurosci. 2018;38(17):4076-92. Epub 2018/03/25. doi: 10.1523/JNEUROSCI.2684-17.2018. PubMed PMID: 29572432.
