# Supplementary material for: Early correction of synaptic long-term depression improves abnormal anxiety-like behavior in adult GluN2B-C456Y-mutant mice
Source: PLoS Biol. 2020 Apr 30;18(4):e3000717. doi: 10.1371/journal.pbio.3000717 (PMC7217483; doi:10.1371/journal.pbio.3000717)

Figure1B. Embryo Whole brain sample

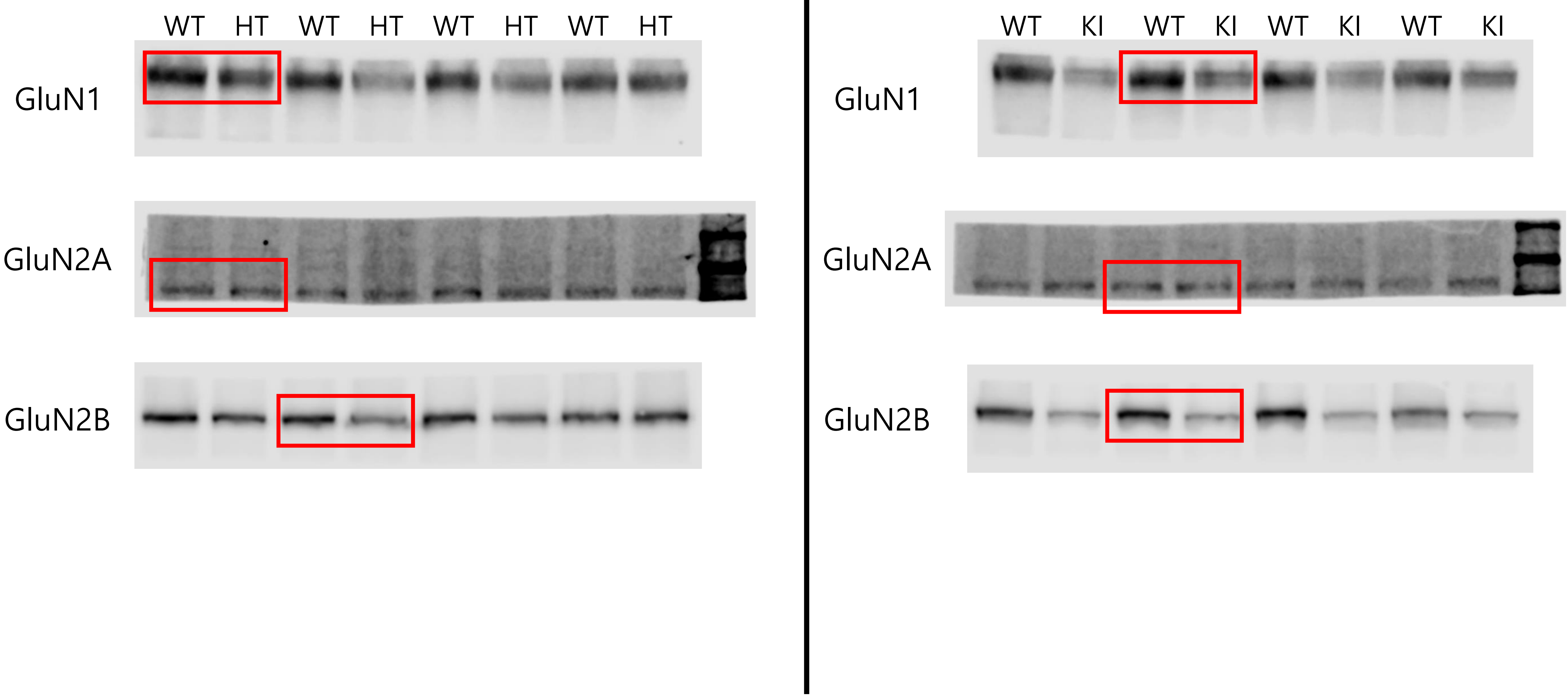

Figure1B. Whole brain fraction sample

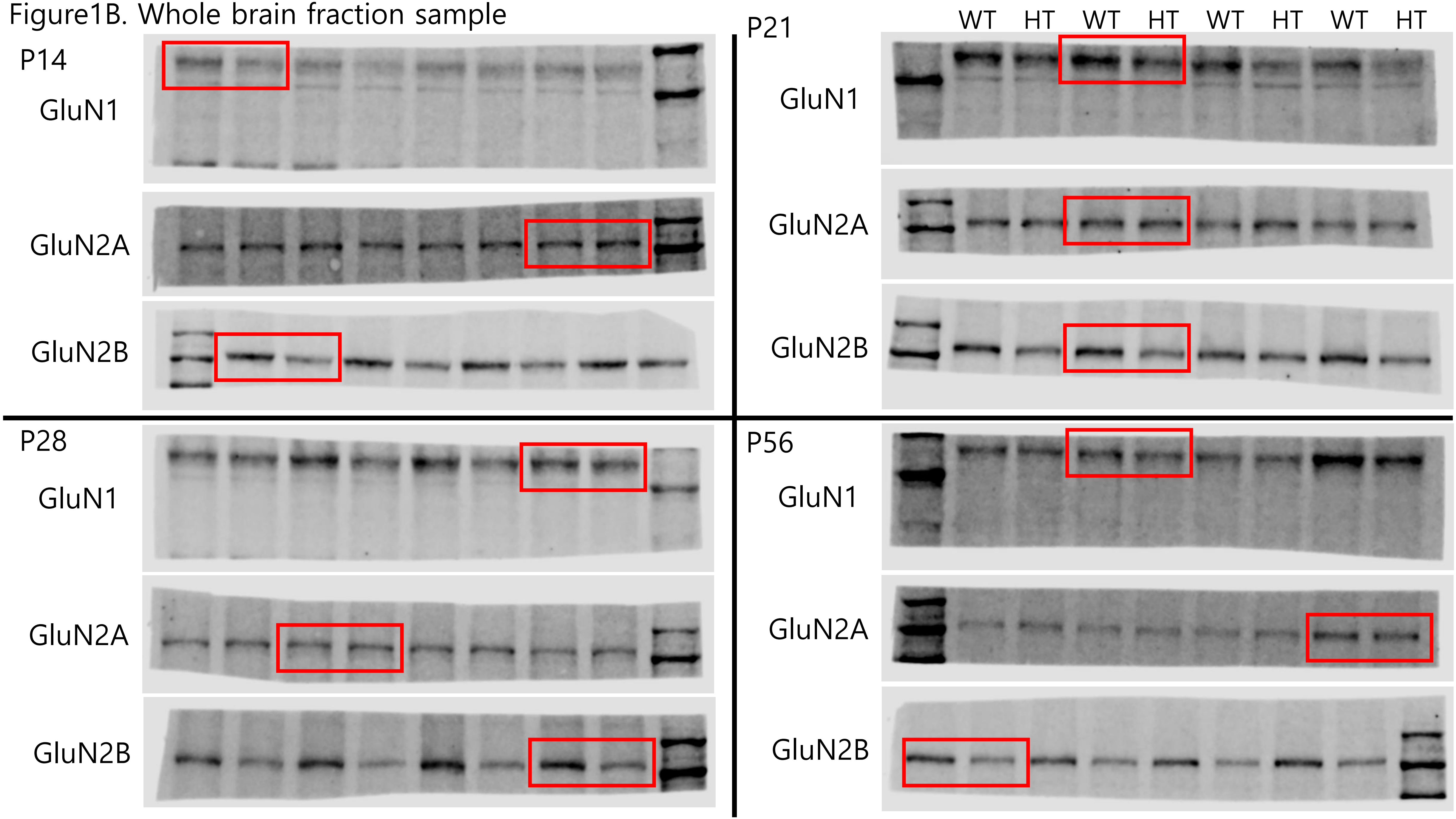

FigureS3B

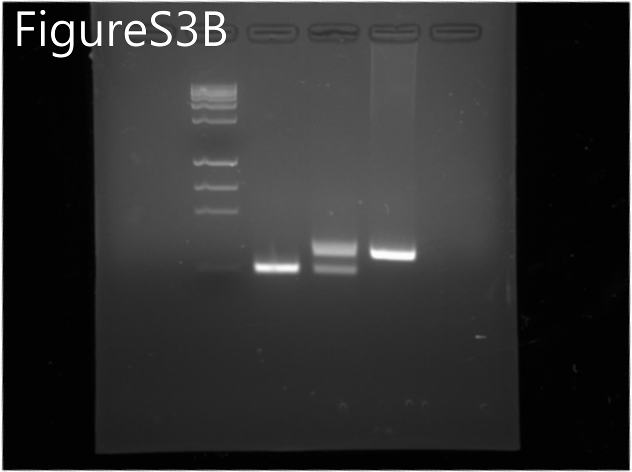

FigureS4A. P2 fraction sample

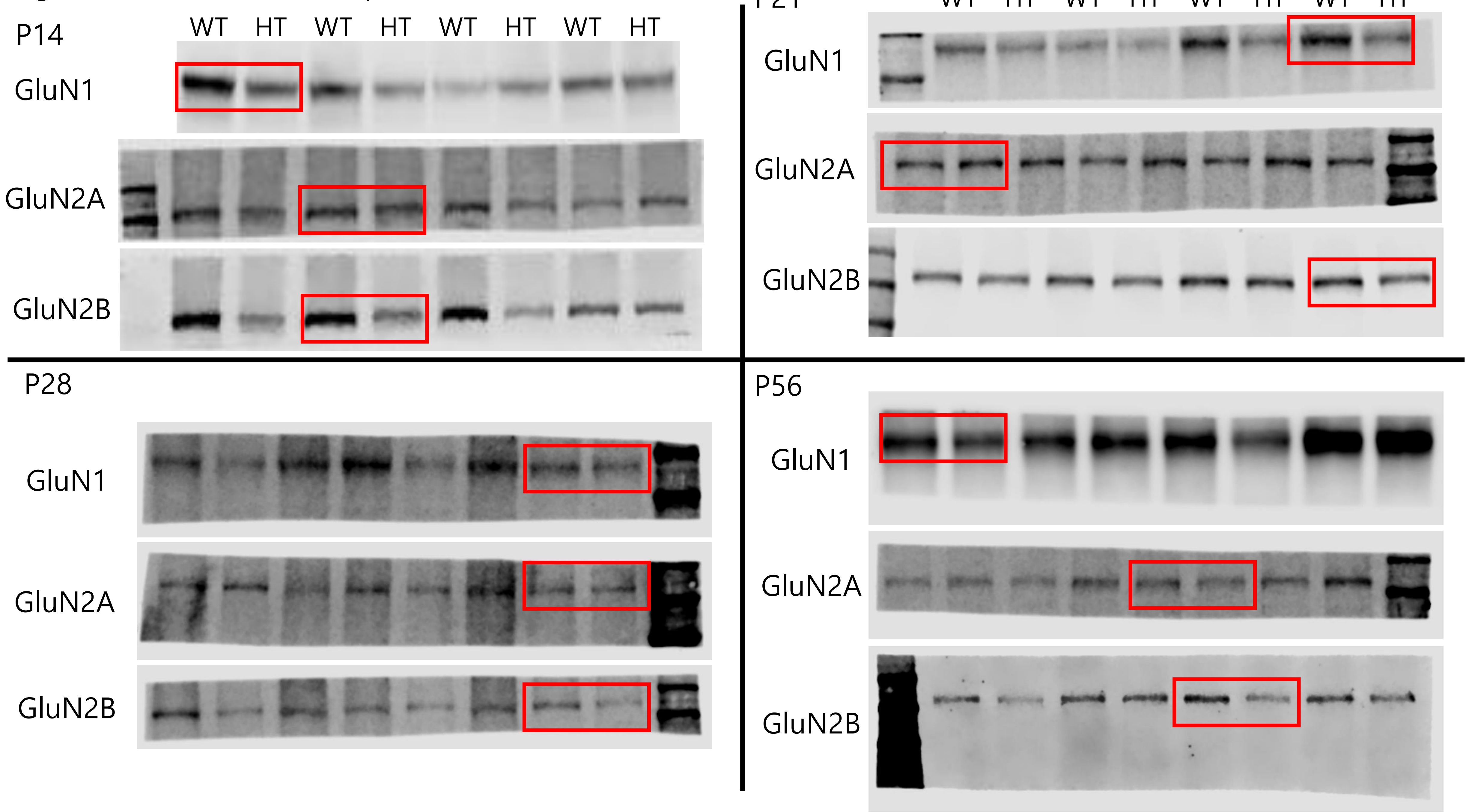

FigureS8A, B. Conventional HT Whole brain fraction sample

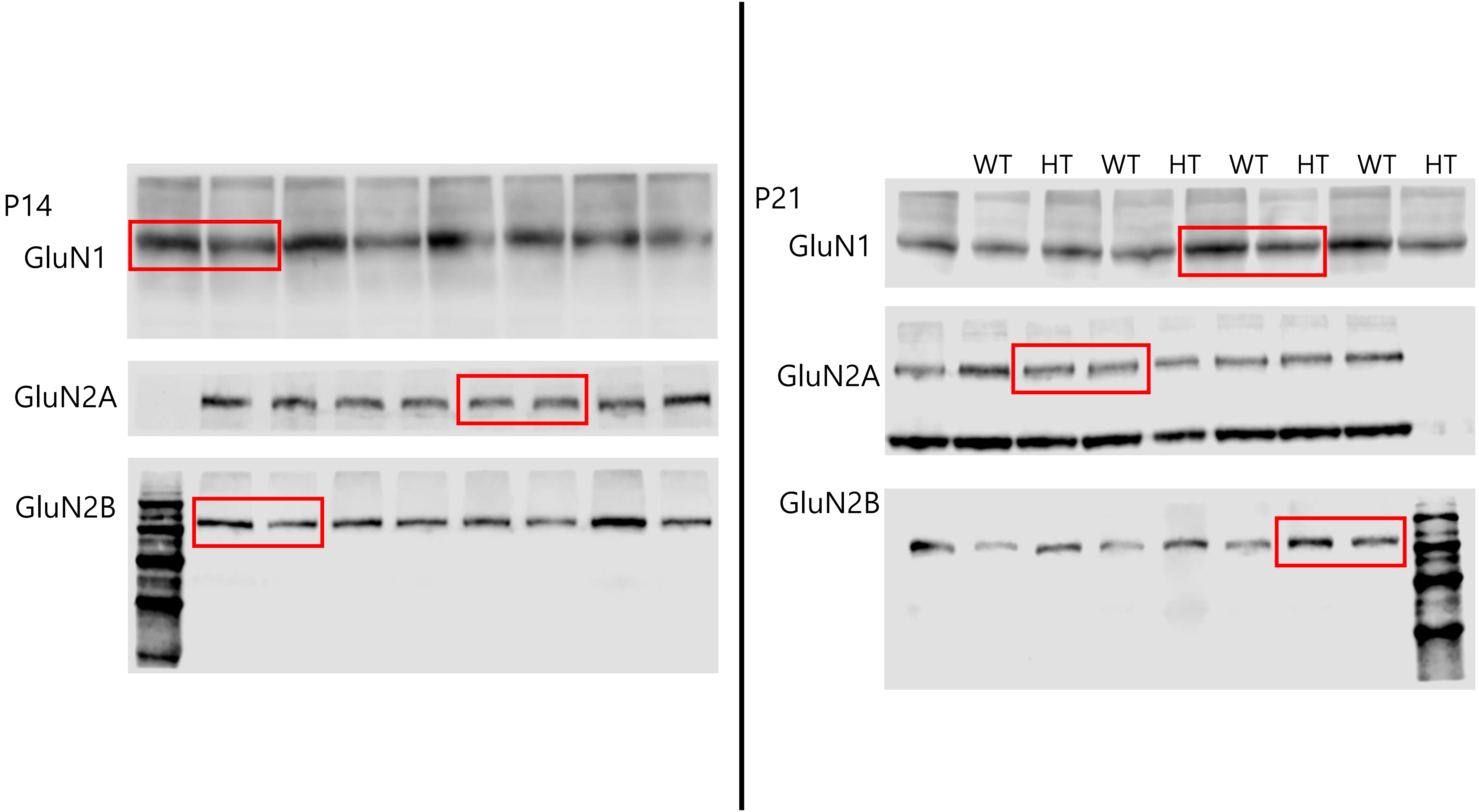

Supplement: S4 Data — (PDF) [file pbio.3000717.s016.pdf]
