# Supplementary material for: Early correction of synaptic long-term depression improves abnormal anxiety-like behavior in adult GluN2B-C456Y-mutant mice
Source: PLoS Biol. 2020 Apr 30;18(4):e3000717. doi: 10.1371/journal.pbio.3000717 (PMC7217483; doi:10.1371/journal.pbio.3000717)
Supplement: Attachment — Submitted filename: Point by point responses to referee comments.docx [file pbio.3000717.s017.docx]

**Point-by-point responses to reviewers’ comment**

**Reviewer #1:** *In the manuscript by Shin et al., the authors developed a mouse model that harbors a C456Y human mutation in the GluN2B subunit. This C456Y mutation is classified as an ASD risk mutation. They perform a systematic analysis of the impact of this mutation on basal synaptic transmission, LTD and LTP and on mouse behavior and report that the mutation likely results in protein misfolding and subsequent degradation. Thus, NMDAR:AMPAR ratios and LFS-induced LTD were reduced. Moreover, the authors report anxiolytic like behavior in the C456Y mutant mice. The synaptic physiology and animal behavior observed in the C456Y mouse can be reversed by chronic administration of D-cycloserine. Unexpectedly and surprisingly, NMDAR-dependent LTP and social interaction behavior were unaltered by the point mutation.
While the generation of a heterozygous mouse line that incorporates the C456Y mutation is novel, the novelty of the results reported here is questionable. Because the mutation results in misfolding and degradation, the synaptic phenotypes and many of the animal behaviors can be phenocopied in the GluN2B heterozygous knockout mouse. The only reported difference between the two mouse models is the degree of self-grooming (increased in the C456Y but not in the het KO). It is unclear what new insights into GluN2B function and/or the identification of roles for sequences of GluN2B that are ASD-relevant are gained by the use of the C456Y mouse. The somewhat surprising finding that early but not late intervention with D-cycloserine was able to reverse the synaptic and behavioral phenotypes is interesting, however, my enthusiasm for these findings is tempered by the following: 1) the partial NMDAR agonist, DCS, has already been shown to reverse synaptic deficits in other animal
models of disease (Na et al., PLOSOne 2017) and has been shown as a potential therapeutic route for ASD. 2) In a VPA model of ASD, Wu et al. (Molecular Neurobiology, 2018), DCS also rescues synaptic and behavioral deficits but through an AMPAR-dependent mechanism. To increase the novelty and provide new insight, it would be important to, in addition to reporting the impact of DCS, to investigate the underlying molecular mechanism(s) that occur in young mice compared to older mice.*

🡪 We appreciate the thoughtful comments of the reviewer. We tried our best to improve the novelty and quality of our manuscript by performing the requested experiments (see below) for the last six months or so.

We would like to point out first that the novelty of our manuscript lies in that we used a knock-in mouse line (*Grin2b^C456Y/+^* mice) carrying a patient mutation and that we have extensively analyzed the synaptic and behavioral phenotypes (relevant to ASD) responsive to a pharmacological rescue (using D-cycloserine). We also performed parallel experiments using *Grin2b^+/–^* mice (carrying conventional *Grin2b* heterozygous mutation) and found that these two mouse lines show largely similar synaptic and behavioral abnormalities responsive to D-cycloserine. Many aspects of these conclusions were strengthened by additional experiments.

The phenotypic differences between *Grin2b^C456Y/+^* and *Grin2b^+/–^* mice are indeed small, but to our knowledge, previous studies have not determined the synaptic and behavioral phenotypes of *Grin2b^+/–^* mice relevant to ASD to the extent described in our study. The only related previous study, to the best of our knowledge, was on *Grin2b^+/–^* mice where the authors analyzed LTP (not LTD) at fimbrial-CA3 synapses but not any behaviors (Ito et al., 1997). Our extensive analyses have also identified small but notable phenotypic differences between *Grin2b^C456Y/+^* and *Grin2b^+/–^* mice; i.e. weaker anxiolytic-like behavior in *Grin2b^+/–^* mice (relative to *Grin2b^C456Y/+^* mice), and reduced GluN2B but not GluN1 proteins in *Grin2b^+/–^* mice, which contrasts the concomitant decreases of both subunits in *Grin2b^C456Y/+^* mice (see the new results below).

Regarding the association of DCS and ASD, it is correct that this concept has been around in the field for a while. However, in order to rigorously test the hypothesis that NMDAR dysfunction is indeed important for ASD, under the context that there are no widely accepted key mechanisms for ASD, we need additional studies on multiple mouse lines involving comprehensive phenotypic analyses and rescues. In addition, GluN2B, a subunit of NMDARs, is a protein that is ‘directly’ associated with the NMDAR dysfunction hypothesis; notably, GluN2A has been less frequently associated with ASD.

Mechanistically, our DCS treatment is ‘early’, and this early correction is ‘long-lasting’, distinguishing our study from earlier studies and suggesting a new strategy for ASD treatment. At the molecular, synaptic, and cellular levels, our new EM analysis indicates that the reduced NMDAR currents and NMDAR-LTD in the mutant mice do not accompany any changes in the density or morphology of dendritic spines, representing excitatory synapses (see below), suggesting that the GluN2B-C456Y mutation mainly affects synapse function but not synapse morphology. In addition, we found that early D-cycloserine treatment rescues LTD by normalizing NMDAR currents (see below the new results). In addition, the balance between excitatory and inhibitory evoked synaptic transmission measured in the same neurons was also not altered (see below for the new results), highlighting that NMDAR function is selectively decreased without affecting AMPAR or GABAR function. Lastly, reduced LTD was also found in the mPFC in addition to the hippocampus (**Fig. 2B**), suggesting that the reductions in NMDAR function and NMDAR-dependent LTD are widespread.

*Major comments:
GluN2B activity and NMDAR-dependent activity has been reported to promote synapse formation. Due to impaired LTD, is synapse pruning altered in CA1? Is synapse density impacted by the C456Y mutation?*

🡪 We appreciate this helpful comment and performed an EM analysis on WT and *Grin2b^C456Y/+^* mice (P21) and found that there is no genotype difference in the density and morphology of dendritic spines in the hippocampal CA1 region (**Fig. 2F**). This result is in line with the lack of changes in the frequency of mEPSCs in the *Grin2b^C456Y/+^* hippocampus (**Supplementary Fig. 5A**), and suggest that LTD reduction by ~50% does not affect the density or morphology of excitatory synapses.

*In supplementary Figure 5, the amplitude of mESPCs and sEPSCs recorded from CA1 pyramidal neurons in slices are virtually identical. This is typically not the case as sEPSCs are generally always larger than mEPSCs.*

🡪 We have to point out that mEPSC amplitudes from WT and mutant mice (30–33 pA) are actually smaller than sEPSC amplitudes (35–40 pA), as shown in S1_Data. We apologize for misleading you, which it might have happened because we used different scales for the y axis in the two graphs (mEPSC and sEPSC).

*Excitation/Inhibition balance should be measured from the same cell in order to examine shifts in E/I balance.*

🡪 We measured the ratio of evoked IPSCs and evoked EPSCs from the same neurons in the hippocampal CA1 region and found no genotype difference (**Supplementary Fig. 5E**), in line with the lack of changes in spontaneous excitatory and inhibitory synaptic transmission (m/s E/IPSCs) (**Supplementary Fig. 5A-D**).

*Does D-cycloserine restore NMDAR:AMPAR ratios and what impact does it have on presynaptic properties at excitatory synapses?*

🡪 We performed the requested experiment and found that, at SC-CA1 synapses of *Grin2b^C456Y/+^* mice, early chronic D-cycloserine treatment (P7–16) normalizes the reduced NMDA/AMPA ratio at a juvenile stage (P19–23), without affecting paired-pulse facilitation (**Fig. 4C,E**).

*What impact does D-cycloserine have on the GluN2B+/- mice? Does this treatment restore the synaptic and behavioral deficits observed?*

🡪 We performed the suggested experiments and found that early D-cycloserine treatment normalizes the decreased LTD in *Grin2b^+/–^* mice (**Supplementary Fig. 11A**), similar to the results from *Grin2b^C456Y/+^* mice (**Fig. 4D**). In behavioral analyses, early D-cycloserine treatment did not rescue the hypoactivity in *Grin2b^+/–^* mice (**Supplementary Fig. 11B**), again similar to the results from *Grin2b^C456Y/+^* mice. We could not determine whether D-cycloserine has any effect on the anxiety-like behavior in *Grin2b^+/–^* mice (**Supplementary Fig. 11C**) because the anxiolytic-like behavior in *Grin2b^+/–^* mice was weaker than that in *Grin2b^C456Y/+^* mice, and the small baseline difference between WT and *Grin2b^+/–^* mice became insignificant (between vehicle-treated WT and *Grin2b^+/–^* mice) by the chronic drug treatment procedures (oral, twice/day for 10 days).

*How is DCS exerting its impact on LTD? Is it simply increasing NMDAR activity, promoting synaptogenesis, shifting the LTD activation curve?*

🡪 Our new results mentioned above show that early DCS treatment (P7–16) rescues NMDA currents without affecting presynaptic release in *Grin2b^C456Y/+^* mice (**Fig. 4C,E**), and that *Grin2b^C456Y/+^* mice have normal excitatory synapse density and morphology (**Fig. 2F**). These results suggest that DCS rescues LTD by normalizing NMDAR currents.

*The rationale for using DCS in the results section is not adequate and appears out of nowhere. Rationale should be provided.*

🡪 We clarified the rationale in the Results section. Again, we would like to express our sincere gratitude to the reviewer for the constructive comments.


**Reviewer #2:** *In this manuscript, Shin et al. investigate synaptic phenotypes and behavioral abnormalities associated with a novel genetic mouse model of ASDs. While the NMDAR hypofunction hypothesis is both popular and supported by numerous human genetic association studies, it has not been rigorously explored using rodent models. In this work, the authors generate a mouse with the C456Y point mutation - a de novo mutation from an individual with ASD and ID -  performing a range of synaptic, morphological and behavioral tests on this animal as well as the GRIN2B heterozygote mice. Furthermore, some of these analyses are performed at multiple developmental stages. This massive amount of high-quality data is a strong point of the manuscript, with somewhat reduced novelty (GRIN2B mutants and GRIN2B pharmacological blockade have been examined in the hippocampus) and lack of mechanistic insight at the physiological and behavioral level detracting from it. The discussion is well
written and thoroughly addresses their findings in the context of the large body of previously published work. The statistical analyses are appropriate. In sum, with some further work it could be appropriate for PLOS Biology given: (1) it focuses on a more genetically accurate model for ASD, (2) will be of broad interest to the ASD field and (3) has been performed with high experimental rigor. Several things could be addressed to strengthen its chances:*

🡪 We appreciate the encouraging and balanced remarks of the reviewer. We tried our best to improve the quality of the manuscript based on the review comments.

*Major Points:
1. Solidifying the comparison between GRIN2B heterozygotes vs C456Y mutation is key: this comparison is a strength of the paper, making a strong case for the point mutation presenting as LOF and arguing for GRIN2B haploinsufficiency. Is the PP-LFS similarly altered in the hets?*

🡪 We performed the suggested experiment and found that LTD is similarly reduced in *Grin2b^+/–^* mice and that early D-cycloserine treatment normalizes the decreased LTD in *Grin2b^+/–^* mice (**Supplementary Fig. 11A**), similar to the results form *Grin2b^C456Y/+^* mice.

*2. Given above, can a subset of the biochemical measures be done in the hets (synaptosomal protein) to further strengthen the idea the disease relevant allele and the het are highly similar. Do they both alter GRIN1 levels at the synapse?*

🡪 We performed the suggested experiments and found that, in *Grin2b^+/–^* mice, the levels of GluN2B, but not GluN1 or GluN2A, subunit of NMDARs were decreased by ~50% at P14 and P21 (**Supplementary Fig. 8A,B**), partly similar to the results from *Grin2b^+/C456Y^* mice where both GluN2B and GluN1 levels were decreased (P14 and P21).

*3. It seems that inhibition was left intact during plasticity recordings – is there any chance that loss of GRIN2B function on interneurons altered the strength of induction protocols – is this monitored in any way? Could this explain the lack of phenotype for the LTP protocols (that reduced inhibition during induction overrode the reduced channel function)?*

🡪 We agree with the reviewer that the inhibitory synaptic transmission left unblocked during LTP measurement might affect LTP induction. In response, we performed LTP experiments in the presence of picrotoxin but could not induce normal HFS-LTP due to somewhat heightened excitation of the neurons in our experimental conditions. We have to point out that spontaneous inhibitory synaptic transmissions (mIPSCs and sIPSCs) and neuronal excitability were unaltered in *Grin2b^C456Y/+^* mice (**Supplementary Fig. 5**), suggesting that inhibitory synaptic transmission in the mutant neurons might not have strong impacts on LTP.

*4. How might the observed behavioral phenotypes relate to ASD? I am not looking for direct correspondence because mice are not humans, but how does “anxiolytic”/reduced anxiety or greater exploration fit as a behavioral model for ASD? I thought anxiety was generally increased clinically? The most consistent phenotype is hypoactivity and reduced entrance to the dark arms. Other assays to assess reduced anxiety or increased exploration to help corroborate the current interpretations should be included*.

🡪 We agree with the reviewer that increased anxiety is a common symptom observed in ASD. However, we have to point out that the mutant pups show increased anxiety-like behavior, as shown by increased USV calls upon separation from their mothers. This early anxiety-like behavior seems to be rapidly decreased to normal levels or anxiolytic-like states at juvenile stages, as shown by the normal time spent with re-united mothers in the maternal homing test and increased open-field center time. Adult mutant mice also show anxiolytic-like behavior in elevated plus-maze and normal behaviors in open-field and light-dark tests. Therefore, *Grin2b^C456Y/+^* mice do show anxiety-like behavior at early stages, which seems to be rapidly normalized or reversed at juvenile and adult stages by some compensatory mechanisms. Unfortunately, we could not test whether the increased USVs in the mutant pups (P4–12) could be due to reduced NMDAR function because the D-cycloserine was given to mice during P7–16. We modified the Discussion to clarify these points.

*5. Several issues regarding approaches to “understand the mechanisms underlying the behavioral abnormalities” need to be addressed in some manner, including:
(a) Does the LTD tested in hippocampus serve as a proxy for plasticity in other brain regions OR do you think the rescued plasticity in the hippocampus is specifically driving the anxiolytic behavior? if the former, then this plasticity phenotype should be shown in another brain region that employs NMDAR-dependent LTD. Alternatively, if the latter, then a manipulation of LTD in the hippocampus of WT should generate an anxiolytic behavior.*

🡪 In response, we measured LTD in the medial prefrontal cortex (mPFC), a brain region frequently associated with ASD. We found that, in *Grin2b^C456Y/+^* mice, LFS-LTD is similarly reduced in the prelimbic region (layer 1) of the mPFC of *Grin2b^C456Y/+^* mice (**Fig. 2B**). This result, together with the reduced LTD in the hippocampus (**Fig. 2A**), suggests that LTD is reduced in multiple brain areas.

*(b) Why does the cycloserine treatment cause a long-term alteration in the LTD? Does it cause lasting increases in NMDAR currents with chronic treatment?*

🡪 In response, we performed additional experiments and found that early D-cycloserine treatment normalizes the reduced NMDA/AMPA ratio at SC-CA1 synapses, without affecting paired-pulse facilitation (**Fig. 4C,E**). These results suggest that D-cycloserine rescues LTD by normalizing NMDAR currents.

*(c) In the adult cycloserine treatment that does not alter behavior, is the LTD not rescued as well?*

🡪 We appreciate this comment but have to point out that LTD is very difficult to measure at adult stages (Collingridge et al., 2010).

*Minor Points:
1. The responsiveness of the mutated receptors to a variety of modulators is examined – why not include d-Cycloserine as well (will complement the rescue of LTD shown later).*

🡪 We performed the suggested experiment and show that D-cycloserine remains a partial agonist at mutant GluN2B-C456Y receptors (although with a modest decrease in efficacy; **Supplementary Fig. 2H**). We would also like to point out that we now show, by additional experiments on neurons, that early D-cycloserine treatment can rescue the decreased NMDAR currents in the *Grin2b^C456Y/+^* hippocampus (**Fig. 4C**), as mentioned above.

Please note that we also took the opportunity of additional experiments on recombinant receptors expressed in Xenopus oocytes to increase n number for two assays (relative Po and zinc sensitivity). Overall conclusions are unchanged with the increased n numbers (**Supplementary Fig. 2B,G**).

*2. Figure 1, the first 2 panels in B should be switched to have all of the HT data together.*

🡪 Corrected.

*3. While it makes sense that mGluR LTD is not altered, it is puzzling that other known NMDAR-dependent forms of plasticity are not altered. This should be discussed.*

🡪 It is indeed known that both LTP (HFS and TBS) and LTD require intact NMDAR function (Collingridge et al., 2010; Malenka and Bear, 2004). However, the LTD-specific decrease in our study might be attributable to the loss of GluN2B, a subunit known to be important for LTD, although this notion is controversial as described in Discussion.

*4. What does “suppressed cognition” mean? Please develop more fully. It comes up in both the results and the discussion.*

🡪 We changed “suppressed cognition” to “suppressed recognition of the fact that a dark and closed place is generally safe” in both Results and Discussion.

*5. Not sure why the pup call phenotypes is interpreted as anxiety?*

🡪 It has been shown that manipulations known to increase or decrease anxiety can increase or decrease pup USV calls in rodents (Winslow and Insel, 1991) (now cited in Results).

**Reviewer #3:** *This manuscript explores the effect of an autism-associated mutation on plasticity and behavior in vivo. The authors demonstrated that the heterozygous GluN2B C456Y mutant mice exhibit decreased GluN2B and GluN1 protein levels, diminished GluN2B-containing NMDARs currents, and reduced LFS-LTD. The GluN2B C456Y mice display hypoactive, anxiolytic-like behavior, but largely normal social behaviors in adults. Chronic administration of the NMDAR partial agonist D-cycloserine in early age (P7-16) restored the reduced LFS-LTD in juvenile mice (P16-21) and the anxiolytic-like behaviors in adult mice (>P56). On the other hand, late acute activation of NMDAR had no effect on the anxiolytic-like behavior in adult GluN2B C456Y mice.*

*General comments: 
In this study, the authors generated a heterozygous ASD-risk GluN2B C456Y mutant mice. The mutant mice displayed decreased GluN2B protein levels and suppressed NMDAR-mediated synaptic plasticity. The authors successfully showed early correction of NMDAR-dependent LTD by chemical treatment and restored anxiolytic-like behavior in adult mice. 
This is a data rich paper with elaborate animal studies, and the overall data quality is convincing. However, the following concerns should be addressed.*

🡪 We appreciate the encouraging comments of the reviewer.

*Major comments:

1.      The authors equate the rescue of LTD as being the cause of the behavioural rescue. This might be the case, but isn’t necessarily true. The language should be adjusted to recognize that their drug treatments can rescue LTD and behaviour, but one doesn’t necessarily cause the other.*

🡪 We fully agree with the reviewer. Indeed, our additional experiments indicated that early D-cycloserine treatment, which rescued LTD, also rescued NMDAR currents (**Fig. 4C**), which have many functions in addition to LTD such as regulation of synaptic proteins and gene expression. We thus modified the relevant text in a way to mention both NMDAR currents and LTD in a more cautious manner throughout the manuscript (Abstract, Introduction, Results, and Discussion).

*2.      Why do the authors use a partial agonist? Have they tried a full agonist?*

🡪 It is because D-cycloserine, a partial agonist at the glycine-binding site of NMDARs approved by U.S. FDA for human uses for the last five decades or so, has been shown to have the potential to treat various neurological and neuropsychiatric disorders (Schade and Paulus, 2016) (now cited in the manuscript). We have not tried a full agonist such as glycine or D-serine.

*3.      The most important finding in this study is the early correction of LTD by “chronic chemical treatment” in young mice and the corresponding long-lasting rescue of anxiolytic-like behavior in adult mice. However, although there are some references for using acute treatment of D-cycloserine, it is still confusing as to why the authors use an “acute treatment” in adult mice rather than the chronic treatment to rescue the anxiolytic-like behavior (Figure 5). It is important to address this discrepancy—ideally by showing the results of chronic treatment in adult mutant mice and a discussion of why this does or does not mimic the same treatment in young animals.*

🡪 We performed the suggested experiment (adult chronic D-cycloserine treatment in adult mice) and found that it has no effect on the anxiolytic-like behavior and hypoactivity in *Grin2b^C456Y/+^* mice (**Supplementary Fig. 12**). However, the chronic drug treatment procedure (oral drug administration twice a day for 10 days using a restrainer, unlike the pup situation where pups were gently grabbed) seems to substantially increase anxiety levels in the mutant mice, blunting the baseline difference in EPM (elevated plus-maze) parameters between vehicle-treated WT and HT mice, thus making it impossible to assess the effect of DCS on these parameters.

*4.       Social deficits are major feature of ASD. The mutant mice display anxiolytic-like behavior but largely normal social behaviors. Further discussion would be helpful. Are the authors convinced this mutation affects one phenotype, but not the other (i.e. social ) or might there be issues with the social behavior paradigms examined. Does the patient have a social deficit?*

🡪 We are pretty confident that *Grin2b^C456Y/+^* mice display normal social behavior because we used various assays for social interaction in adult mice (three-chamber social approach, dyad direct social interaction, and ultrasonic courtship social communication) and juvenile mice (interaction with reunited mothers in the maternal homing test). However, we agree with the reviewer that the social tests and parameters that we employed in the present study may not be sensitive enough to detect certain social deficits. We commented on this point in Discussion. A previous study on this GluN2B-C456Y mutation provided little information other than the fact the mutation was identified from a male individual with autism and intellectual disability (O'Roak et al., 2012).

*5.      Supplementary Figure 1 and 2 provide a nice mechanistic explanation for the severity of the GluN2B C456Y mutation. The supplementary data could be included in the main figures. Otherwise, the text for the supplementary figure 1 and 2 should be resized.*

🡪 Because our paper mostly focuses on in vivo mouse analysis and because, the contents in **Supplementary Fig. 1 and 2** have been partly reported previously, as indicated in Results, we prefer to keep these data in the Supplementary material. The description for these two figures are important for the understanding of the function of the mutation and is already pretty condensed in our opinion; your understanding would be much appreciated.

*6.      The resolutions for all the figures could be improved. The authors should check the image size and resolution in the revised version.*

🡪 We tried to improve the size and resolution of the images in the revised manuscript.

**Reviewer #4:** *In the present manuscript by Shin, et al., the authors have generated a KI mouse with a human autism spectrum associated de novo mutation in GluN2B, the C456Y mutation. The GluN2B gene is a frequently mutated risk gene in autism and understanding the effects of these strong de novo mutations in animal models is very timely and important. Appropriately, the authors primarily looked at the heterozygous KI mice, which is consistent with the disease expression. In the current study, the authors have combined biochemistry, electrophysiology, and behavioral analyses to examine the effects of this mutation. In general, very little novel information was found in the extensive study. Consistent with two prior studies, the expression of the GluN2B-C456Y mutant is significantly impaired, possibly through a folding or forward trafficking defect as the mRNA levels are normal. This expression deficit affected NMDA receptor levels, caused a modest reduction in LTD expression, and had some modest effects on behavior in the het mice. These findings are in line with what has been observed in het 2B KO. More importantly, the authors have showed some ability to at least partially rescue these modest deficits by an early live treatment with D-cycloserine, an NMDAR glycine-site partial agonist. Obviously, since a major goal in understanding how these de novo mutants cause autism spectrum disorders is to develop treatments, this is the major finding of this study. Overall, I don’t think there are any new insights in this paper, but it is nonetheless an important contribution to the literature.  Some minor comments listed below:*

🡪 Thanks for the thoughtful comments of the reviewer. We tried to improve the manuscript by performing additional experiments suggested by the reviewers for the last six months or so.

The GluN2B-C456Y mutation does decrease protein levels in heterologous cells, but our work demonstrates that this mutation can lead to decreased protein levels in vivo. In addition, our results indicate that GluN1 is also decreased in addition to GluN2B in *Grin2b^C456Y/+^* mice, whereas only GluN2B but not GluN1 is decreased in *Grin2b^+/–^* mice carrying conventional heterozygous *Grin2b* mutation (found during revision) (**Supplementary Fig. 8A,B**). This is one of the few examples of distinct changes observed in *Grin2b^C456Y/+^* and *Grin2b^+/–^* mice, although these mice show largely similar synaptic and behavioral changes.

We have to point out that LTD and related behaviors were not measured in *Grin2b^+/–^* mice in any previous studies, as described in the Introduction. The only study that reported any type of changes in synaptic plasticity was the one that reported LTP (not LTD) in the fimbrial-CA3 synapse (Ito et al., 1997), and this study did not explore any behavioral changes. Therefore, our study is the first to our knowledge that attempted comprehensive and comparative analyses of both synaptic and behavioral phenotypes that are responsive to pharmacological rescues in two heterozygous *Grin2b* mouse lines (*Grin2b^C456Y/+^* and *Grin2b^+/–^*), providing solid evidence for that the GluN2B-C456Y mutation causes loss-of-function phenotypes in vivo (see our new data in **Supplementary Fig. 11, 12**).

We appreciate the reviewer’s point on the importance of the pharmacological rescue under the context of ASD. Adding more, our study is unique in that we attempted an ‘early’ rescue of the key pathophysiology, and also demonstrated that the effects are ‘long-lasting’, standing uniquely among many other studies that tried drug-dependent temporary reversal of behavioral deficits in adult mice.

Lastly, we have performed many additional experiments adding more insights into the molecular, synaptic, and cellular features of our findings. Mentioning the main ones, the GluN2B-C456Y mutation has no effect on excitatory synapse density and morphology, as supported by EM analyses (**Fig. 2F**) or the balance between synaptic excitation and inhibition (**Supplementary Fig. 5E**), suggesting that the mutation mainly affects NMDAR function but not synapse structure or AMPAR/GABAR function. We also found that early chronic D-cycloserine treatment rescues LTD by normalizing NMDAR currents without affecting presynaptic release (**Fig. 4C**), suggesting that D-cycloserine may rescue abnormal behaviors not only through LTD but also through changes occurring downstream of NMDAR activation such as protein phosphorylation and gene expression.

*1)      The lack of an effect on LTP could be a consequence of looking at a developmental age (P27-P33) when the switch from synaptic GluN2B to GluN2A is largely complete, thus minimizing the effect of impaired GluN2B expression.*

🡪 We appreciate this point, and mentioned it Discussion.

*2)      So many behavioral experiments were performed, I am worried this constitutes a battery of test looking for an effect, and thus significance would need to be corrected for multiple comparisons. If this correction were made, I suspect all of the effects would be insignificant.*

🡪 We agree with the reviewer that all of the behavioral changes we found statistically significant may become insignificant after correcting for multiple comparisons.

Although conducting multiple tests without an adjustment for multiplicity can increase chance findings (Type 1 error), the adjustment for multiplicity may also inflate the probability of Type 2 error, resulting in increased chances of missing an effect when there is actually one. This can be a drawback of multiple testing adjustment for studies where a variety of behaviors are tested. For this reason, we would like to report the results without multiple comparison adjustments.

*3)      The effects on LTD were modest and it was not blocked. So language throughout should reflect this more accurately. For example, top of page 18, “strongly reduced LFS-LTD” is overstated.*

🡪 We have to point out that we analyzed heterozygous *Grin2b^C456Y/+^* mice, where LTD was reduced by ~55%; homozygous mutant mice could not be tested for LTD for embryonic lethality. However, we agree with the reviewer that results should be carefully described and changed the text from “…strongly reduced NMDAR-dependent LFS-LTD...” to “…reduced NMDAR-dependent LFS-LTD by ~55%...”. In addition, we tried not to exaggerate the extent of changes in LTD throughout the manuscript.

We would also like to point out that, during revision, we observed a decrease in LTD in the medial prefrontal cortex in addition to the hippocampus, and the extent of the decrease was greater (~84%) than that in the hippocampus (**Fig. 2B**). In addition, this result suggests that decreased LTD may a widespread change occurring in different brain regions of *Grin2b^C456Y/+^* mice.

*4)      Figure 1: not sure the sequencing data is necessary. Also the arrangement of the data in B does not facilitate rapidly seeing the data. Vertical is generally better, and the homozygous data should either be first or last.*

🡪 We believe that showing the sequencing data might help readers in perceiving that our study is on a knock-in mouse line carrying a patient-derived mutation. We also re-organized the images in **Fig. 1B**, as suggested.

*5)      Various typos and possible language-based mistakes throughout that would benefit from careful editing.*

🡪 We carefully corrected the text throughout the manuscript.

*6)      Page 6: ~20% “increase” – I think this should be decrease.*

🡪 It is actually an increase in Po (as revealed by the decrease in the value of the MK-801 inhibition tau on). In this assay, the faster the MK-801 inhibition is, the higher the channel Po is.

**References**

Collingridge, G.L., Peineau, S., Howland, J.G., and Wang, Y.T. (2010). Long-term depression in the CNS. Nature reviews Neuroscience *11*, 459-473.

Ito, I., Futai, K., Katagiri, H., Watanabe, M., Sakimura, K., Mishina, M., and Sugiyama, H. (1997). Synapse-selective impairment of NMDA receptor functions in mice lacking NMDA receptor epsilon 1 or epsilon 2 subunit. The Journal of physiology *500 ( Pt 2)*, 401-408.

Malenka, R.C., and Bear, M.F. (2004). LTP and LTD: an embarrassment of riches. Neuron *44*, 5-21.

O'Roak, B.J., Vives, L., Fu, W., Egertson, J.D., Stanaway, I.B., Phelps, I.G., Carvill, G., Kumar, A., Lee, C., Ankenman, K.*, et al.* (2012). Multiplex targeted sequencing identifies recurrently mutated genes in autism spectrum disorders. Science *338*, 1619-1622.

Schade, S., and Paulus, W. (2016). D-Cycloserine in Neuropsychiatric Diseases: A Systematic Review. The international journal of neuropsychopharmacology / official scientific journal of the Collegium Internationale Neuropsychopharmacologicum *19*.

Winslow, J.T., and Insel, T.R. (1991). The infant rat separation paradigm: a novel test for novel anxiolytics. Trends Pharmacol Sci *12*, 402-404.
