# Supplementary material for: Early correction of synaptic long-term depression improves abnormal anxiety-like behavior in adult GluN2B-C456Y-mutant mice
Source: PLoS Biol. 2020 Apr 30;18(4):e3000717. doi: 10.1371/journal.pbio.3000717 (PMC7217483; doi:10.1371/journal.pbio.3000717)
Supplement: Attachment — Submitted filename: Response to reviewers Shin et al.docx [file pbio.3000717.s019.docx]

**Response to reviewers**

***Reviewer #1:*** *The authors have adequately addressed the majority of this reviewer's concerns. With the significant changes made to the manuscript during the revision, the paper is suitable for publication.*

We appreciate the final comments of the reviewer.

***Reviewer #2, Marc Fuccillo:*** *The authors have attempted to address many of my requests. In doing so, they have strengthened the idea that the C456Y point mutant functions like the NR2B heterozygous LOF mutation, both at the synaptic, protein and behavioral level. While perhaps expected, these data provide clarity to the role of ASD-associated NMDAR mutations. Another strong plus is the addition of data showing a similar plasticity effect in the mPFC. these data suggest that the data in hippo are a proxy for other areas (something that should be mentioned in the text). 

Overall, I feel like this is a strong contribution to the asd pathophysiology literature. while not incredibly surprising, the data is clear and makes a strong point supporting this specific disease-associated mutation as GRIN2B haploinsufficiency. the reproducibility of DCS rescue across assays is also a strength.*

We appreciate the final comments of the reviewer. We agree with the possibility that the hippocampus may be a proxy for other brain areas (see below); we added these points to Discussion. *A few short textual things should be added:
1. discuss the relationship between anxiogenic and anxiolytic behaviors as they relate to ASD. these are not part of the core behavioral phenotype but are the most clearly altered in this work. the literature on ASD and anxiety-related behaviors should be discussed.*

We discussed the relationship between anxiogenic and anxiolytic behaviors with related literature on ASD and anxiety-related behaviors.

*2. please discuss that in light of the mPFC data, it is unclear which brain regions are contributing to the mutation-associated behavioral (and rescue-related) changes.*

The similar decreases in LTD in the hippocampus and the mPFC suggest that the hippocampus may be a proxy for other brain areas and that many brain areas may be associated with the behavioral phenotypes caused by the *Grin2b* mutation and haploinsufficiency. We made these points in Discussion.

*3. "suppressed recognition of the fact that a dark and closed place is generally safe" - this isn't any better than before - perhaps just describe the phenotype.*

In response, we deleted this conjecture and just described the phenotype in Results.

***Reviewer #3:*** *The authors performed extra experiments and gave explanations to address all the questions raised by the reviewers. The manuscript became even more data heavy and the conclusions are now better discussed to support the authors ideas. Although there are some technical issues, the authors do demonstrate GluN2B-C456Y haploinsufficiency decreases GluN2B protein levels, LTD, and anxiety-like behavior. The overall behavioral and physiological experiments provide an insight for the importance of early correction of pathophysiological deficits.
The findings are important in the field for the treatment of neurodevelopmental or psychiatric diseases caused by NMDAR mutations.*

We appreciate the final comments of the reviewer.

***Reviewer #4:*** *Satisfied with the manuscript as edited. Again, it is an important contribution to the literature as I stated in my initial review, though there are not major new insights here. Addition of the PFC data at least provides some additional evidence for more general deficits.*

We appreciate the final comments of the reviewer.
